# The Antidepressant Sertraline Affects Cell Signaling and Metabolism in *Trichophyton rubrum*

**DOI:** 10.3390/jof9020275

**Published:** 2023-02-20

**Authors:** Flaviane M. Galvão-Rocha, Carlos H. L. Rocha, Maíra P. Martins, Pablo R. Sanches, Tamires A. Bitencourt, Matthew S. Sachs, Nilce M. Martinez-Rossi, Antonio Rossi

**Affiliations:** 1 Department of Genetics, Ribeirao Preto Medical School, University of São Paulo, USP, Ribeirao Preto 14049-900, SP, Brazil; 2 Department of Biology, Texas A&M University, College Station, TX 77843-3258, USA

**Keywords:** RNA-seq, drug repositioning, dermatophyte, membrane damage, oxidative stress, ergosterol, sertraline, *Trichophyton rubrum*

## Abstract

The dermatophyte *Trichophyton rubrum* is responsible for most human cutaneous infections. Its treatment is complex, mainly because there are only a few structural classes of fungal inhibitors. Therefore, new strategies addressing these problems are essential. The development of new drugs is time-consuming and expensive. The repositioning of drugs already used in medical practice has emerged as an alternative to discovering new drugs. The antidepressant sertraline (SRT) kills several important fungal pathogens. Accordingly, we investigated the inhibitory mechanism of SRT in *T. rubrum* to broaden the knowledge of its impact on eukaryotic microorganisms and to assess its potential for future use in dermatophytosis treatments. We performed next-generation sequencing (RNA-seq) to identify the genes responding to SRT at the transcript level. We identified that a major effect of SRT was to alter expression for genes involved in maintaining fungal cell wall and plasma membrane stability, including ergosterol biosynthetic genes. SRT also altered the expression of genes encoding enzymes related to fungal energy metabolism, cellular detoxification, and defense against oxidative stress. Our findings provide insights into a specific molecular network interaction that maintains metabolic stability and is perturbed by SRT, showing potential targets for its strategic use in dermatophytosis.

## 1. Introduction

Dermatophytoses are skin infections caused by fungi. These dermatophytes are associated with keratinized tissues, such as skin, hair, and nails. Although dermatophytes do not usually cause deep lesions, the disease can progress to severe acute illness in hosts with poor immune protection [1]. *Trichophyton rubrum* is responsible for most onychomycosis cases in humans worldwide [2].

The number of antifungal agents available for the treatment of dermatophyte infections is limited. The most used drugs converge to a similar mechanism of action in fungal cells, targeting ergosterol biosynthesis. Consequently, intense selective pressure is exerted on clinical isolates, leading to the emergence of strains that are resistant to available drugs [3,4].

The development of new antifungal drugs is limited by the similarities between fungal and host cells, which restrict the number of cellular targets [4]. Furthermore, the time-consuming efforts and elevated costs required to achieve effective drugs engender the need to exploit alternatives for the management of fungal infections [4,5]. One approach is to evaluate the potential use of non-antifungal drugs as antifungals [6].

Numerous drugs used to treat non-infectious conditions such as inflammation, cardiovascular diseases, or depression are known to exhibit antimicrobial activities. The selective serotonin reuptake inhibitor (SSRI) sertraline (SRT), commonly used as an antidepressant, was revealed to have antifungal activity [7,8,9,10]. Furthermore, this drug interacts synergistically in vitro with antifungals such as azoles, caspofungin, and amphotericin B, indicating its potential use as an adjuvant in treating fungal infections [11,12,13,14,15]. This synergistic association could decrease drug dosages and emerging drug resistance rates [6].

In the mammalian nervous system, SRT inhibits serotonin (5-hydroxytryptamine, 5-HT) reuptake, releasing serotonin into the synaptic cleft [16]. In addition, the drug enhances 5-HT synthesis by increasing the expression of tryptophan hydroxylase, the enzyme responsible for converting L-tryptophan to 5-HT [17]. SRT targets the serotonin transporter (5-HTT) responsible for 5-HT reuptake into presynaptic neurons in human cells. Interestingly, there is no conserved homolog of the 5-HT transporter in fungi, highlighting the occurrence of secondary targets for this drug [11]. However, the mechanisms underlying the antifungal activity of SRT remain unclear. It has been proposed that SRT intercalates into the phospholipid membranes of V-ATPase-acidified organelles [18]. Moreover, it is possible that the potent antifungal activity against *Cryptococcus neoformans* could also be driven by the inhibition of protein synthesis and/or other actions interfering with lipid metabolism [10,11].

Here, we evaluated the effects of sub-lethal doses of SRT against the dermatophyte *T. rubrum* through RNA-seq analysis. Our findings revealed changes in the comprehensive induction of the cellular antioxidant system, membrane transport-associated genes, and biosynthetic genes, resulting in changes in the cell membrane. 

## 2. Materials and Methods

### 2.1. Strain and Culture Conditions for RNA-Seq Analysis

*T. rubrum* strain CBS118892 from the Westerdijk Fungal Biodiversity Institute (formerly CBS-KNAW Collections), The Netherlands, was maintained on malt extract agar (2% glucose, 2% malt extract, 0.1% peptone (*w/v*), pH 5.7). Conidial suspensions were obtained by flooding 21-day-old plates with sterile 0.9% NaCl, recovering the liquid, mixing by vortexing, and filtering through glass wool. We estimated the conidial concentration by counting in a Neubauer chamber. Approximately 1 × 10^6^ conidia were inoculated into 100 mL Sabouraud dextrose broth (SDB; 2% (*w/v*) glucose, 1% (*w/v*) peptone) and incubated with agitation (120 rpm) at 28 °C for 96 h. Next, we aseptically transferred mycelia into new flasks containing 100 mL SDB media with 70 µg/mL of SRT hydrochloride (Cayman Chemical Co., Ann Arbor, MI, USA), corresponding to 70% of the minimal inhibitory concentration (MIC) [15], and in the absence of drugs (control). After 3 or 12 h of incubation at 28 °C with agitation, the resultant mycelia were collected, quick-frozen, and stored at −80 °C until RNA isolation.

### 2.2. RNA Isolation and cDNA Library Construction

Total RNA was isolated from ~100 mg mycelia using the Illustra RNAspin mini RNA isolation kit (GE Healthcare, Chicago, IL, USA). RNA concentrations were determined using a NanoDrop ND-1000 spectrophotometer (Thermo Fisher Scientific, Waltham, MA, USA). RNA quality was verified by agarose electrophoresis and an Agilent 2100 Bioanalyzer (Agilent Technologies, Santa Clara, CA, USA). Equal amounts of RNA from three independent biological replicates at each time point (3 and 12 h) were used for cDNA synthesis using the TruSeq RNA library kit (Illumina, San Diego, CA, USA) and sequenced using Illumina technology (HiSeq 2000 sequencer) according to the manufacturer’s instructions. Paired-end reads were 150 bp in size.

### 2.3. Transcriptome Analysis

Raw read data obtained were filtered for quality control using the FastQC tool and trimmed using Trimmomatic [19] to remove adapters and Illumina-specific sequences. Trimmed paired-end reads from each sample were aligned to the reference genome using the STAR aligner [20]. We inspected the average coverage of transcripts and alignments using the Interactive Genomics Viewer (IGV) software [21]. We generated gene-level read counts by STAR using the quantModeGeneCounts option. Differential expression was analyzed using the DESeq2 Bioconductor package [22]. 

After the above, the Benjamini–Hochberg correction was applied (*p* < 0.05) [23], and a cutoff threshold of ±1.5 log2 fold change was set to reveal statistically significant expression differences (genes surpassing these thresholds are referred to as differentially expressed genes (DEG)) [24]. We functionally categorized them using Gene Ontology (GO) terms assigned by the Blast2GO algorithm. Highly represented categories were determined by enrichment analysis using the BayGO algorithm [25].

### 2.4. Data Analysis and Gene Selection

We predicted transporter-associated domain-containing proteins in the *T. rubrum* CBS 118892 genome sequence using the HMMER v3.1 b2 pipeline [26]. We built the hidden Markov model (HMM) utilizing a Pfam [27] multiple alignment-based search of 55 sequences corresponding to the ABC domain-containing proteins and another Pfam multiple alignment-based search of 192 sequences corresponding to the MFS (major facilitator superfamily) domain-containing proteins, both from different organisms (https://pfam.xfam.org/family/PF00005 and https://pfam.xfam.org/family/PF07690, respectively—accessed on 10 June 2022). The resulting models with epitopes of the consensus sequences of transporter-associated domain-containing proteins were used to search for homologs in *T. rubrum* protein sequences. In addition, we identified proteins using the keywords “transporter”, “transcription factor”, and “kinase” in Ensembl Fungi (https://fungi.ensembl.org). We compared the gene codes for specific proteins with the DEGs identified in RNA-seq.

### 2.5. cDNA Synthesis and RT-qPCR Analysis

We treated total RNA with DNase I (Sigma-Aldrich, Milwaukee, WI, USA) to remove residual genomic DNA and generate complementary DNA (cDNA) using a High-Capacity cDNA Synthesis Kit (Applied Biosystems, Foster City, CA, USA). We performed RT-qPCR with a StepOnePlus Real-Time PCR system (Applied Biosystems). Reactions were run in 12.5 μL with Power SYBR Green PCR Master Mix (Applied Biosystems), 70 ng template cDNA, and forward and reverse primers (Table S1). We used glyceraldehyde-3-phosphate dehydrogenase (*gapdh*) and DNA-dependent RNA polymerase II (*rpb2*) as internal controls [28]. Thermocycler conditions included an initial PCR step at 95 °C for 10 min, followed by 40 cycles at 95 °C for 15 s and 60 °C for 1 min. Data were obtained from three independent replicates. The 2^−∆∆ct^ relative expression quantification method was used to calculate gene responsiveness [29]. For RNA-seq validation, we used 3 h and 12 h without SRT as the reference samples. Statistical analyses were performed using a *t*-test followed by Tukey’s post-hoc test using GraphPad Prism v. 5.1 software (GraphPad, La Jolla, CA, USA). A significance level of 95% was considered; therefore, the measures were statistically different at *p* < 0.05.

### 2.6. Quantitation of Ergosterol Content

The *T. rubrum* strain CBS118892 was cultivated on Sabouraud dextrose agar (SDA) for 17 days at 28 °C. The mycelia were transferred to 50 mL of SDB for 24 h at 28 °C with agitation (200 rpm). After this period, the mycelium was recovered, inoculated in 20 mL of SDA containing a sub-inhibitory concentration of SRT (6.25 µg/mL), ketoconazole (KTC, 4 µg/mL; Sigma-Aldrich), or amphotericin B (AMB, 1.25 µg/mL; Sigma-Aldrich), and incubated for 48 h at 28 °C, 200 rpm. Mycelia were also inoculated in SDA without drugs (control). The resulting mycelia were filtered, the dry weight was measured, and the ergosterol content was determined following the protocol described by Arthington-Skaggs et al. [30], with modifications [31]. Briefly, 3 mL of 25% alcoholic potassium hydroxide solution was added to the dried mycelia and mixed by vortexing for 5 min. We incubated the mycelium at 85 °C for 1 h. The tubes were cooled to room temperature and sterile distilled water (1 mL) and 3 mL of *n*-heptane were added, followed by vigorous vortex mixing for 5 min. The heptane layer was transferred to a clean polystyrene tube, and a 20 μL aliquot was diluted five-fold in 100% ethanol and scanned in a spectrophotometer between 230 and 300 nm. All assays were performed in triplicate. Both 24(28)-dehydroergosterol (a sterol pathway intermediate) and ergosterol absorb at 281.5 nm, but 24(28)-dehydroergosterol also absorbs at 230 nm, which was subtracted from the absorbance obtained at 281.5 nm [31]. We calculated the reduction in ergosterol content as the difference in the treatments compared to the growth control samples based on a standard curve of various concentrations of standard ergosterol (Sigma-Aldrich). The results are expressed as a percentage of ergosterol content compared with the growth control. One-way ANOVA was used to determine the amount of ergosterol in the control with the treatments, followed by Tukey’s post-hoc test. Statistical significance was set at *p* < 0.05, and Prism v. 5.1 software was used to generate graphs and perform statistical analyses.

## 3. Results 

### 3.1. Transcriptional Profiling of Trichophyton rubrum Challenged by SRT

**Figure 1:**
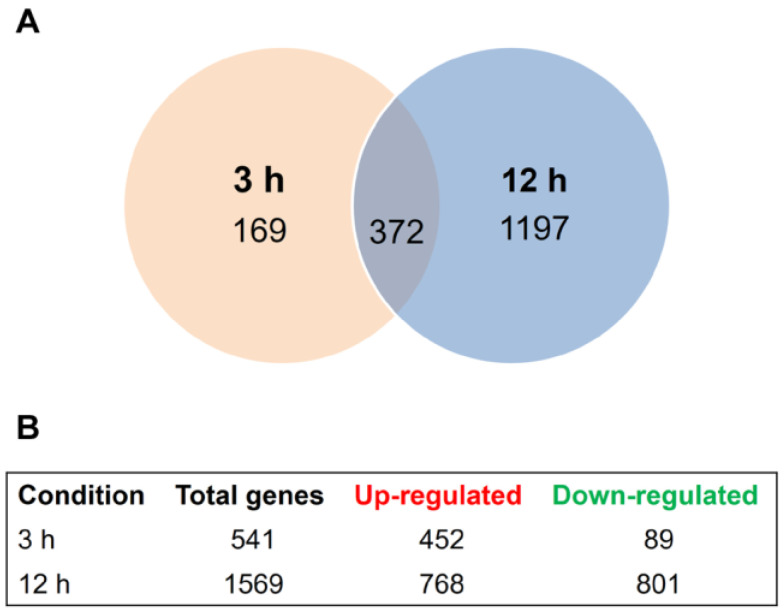
SRT-induced differential gene expression. (**A**) Venn diagram presenting the time-dependent differential expression of 1738 genes after exposure to sub-lethal dose of SRT for 3 h and 12 h compared to the control samples without SRT. (**B**) Total up- and downregulated genes in each experimental condition.

We used next-generation sequencing to comprehensively analyze the *T. rubrum* global transcriptome in response to sub-lethal doses of the antidepressant SRT at two different time points. Approximately 535.5 million high-quality reads of 150-bp paired-end sequences were obtained (Table S2). In response to SRT exposure, 169 and 1197 genes were modulated at 3 and 12 h, respectively, when compared to the expression levels of the control. Three hundred seventy-two genes were modulated at both time points, totaling 1738 genes that were responsive to SRT (Figure 1A). SRT differentially upregulates more genes than it represses after 3 h of treatment. This profile was inverted at 12 h exposure with a less expressive difference between up- and downregulated genes compared to the 3 h time point (Figure 1B). We list the products of all genes modulated in response to SRT for each drug exposure time in Table S3 and present the most significantly up- and downregulated genes in Table 1.

**Figure 2:**
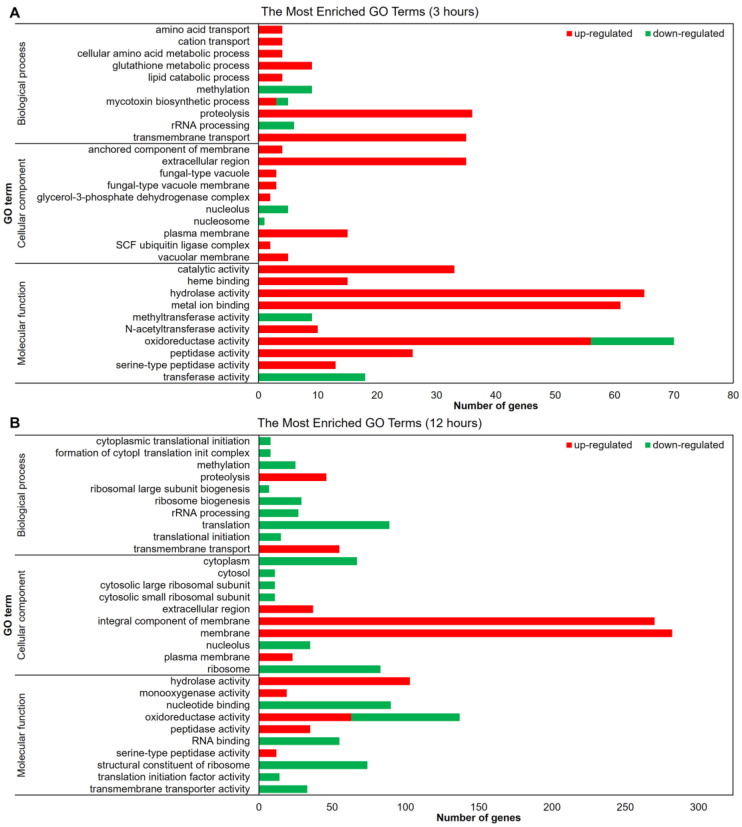
Gene ontology-based functional categorization of the most representative differentially expressed genes. The red and green bars indicate the number of up- and downregulated genes modulated by *T. rubrum* in response to SRT (*p* < 0.05). Results show genes after (**A**) 3 h and (**B**) 12 h drug exposure compared to the control without SRT.

The functional categorization of the modulated genes was performed using Blast2Go, which revealed the molecular mechanisms by which *T. rubrum* senses and responds to the challenge of SRT presence (Figure 2). Gene function analysis using GO enriched mainly molecular function terms after 3 h of treatment. Genes associated with catalytic and hydrolase activities and those related to transmembrane transport were upregulated. The oxidoreductase activity induces or represses many genes after 3 h of drug exposure (Figure 2A). The upregulated membrane-associated genes and downregulated genes associated with the oxidation–reduction process increased at 12 h compared to those after 3 h of treatment. The terms related to translation were all inhibited at the 12 h time point. The number of genes associated with hydrolase activity and transmembrane transport increased compared to those in the 3 h condition, remaining induced in response to 12 h of SRT exposure (Figure 2B).

**Figure 3:**
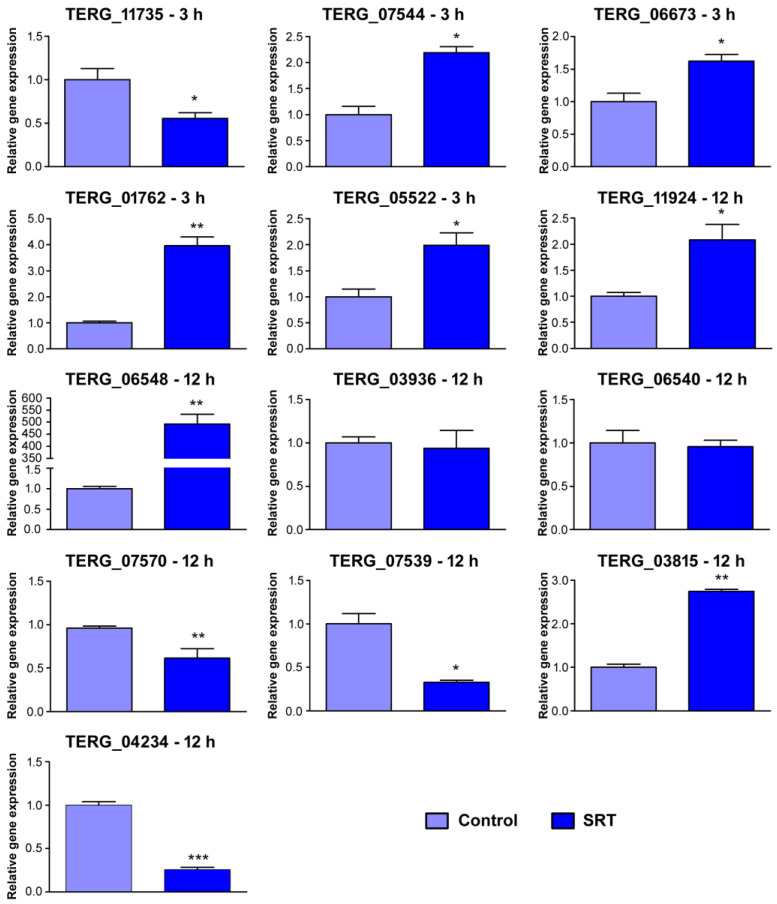
Validation by RT-qPCR of 15 genes differentially expressed in response to SRT exposure. Log2 fold change at each time point represents gene expression level changes at each time point. Expression levels are relative to the control without SRT. Asterisks indicate the statistical significance determined by the *t*-test followed by Tukey’s post-hoc test (* *p* < 0.05; ** *p* < 0.01; *** *p* < 0.001). The identification of each gene is listed in Table 2.

To validate the RNA-seq results, we randomly selected 15 genes from the differentially expressed genes related to processes such as catalytic activity, transferase activity, and transmembrane transport (Figure 3). The RT-qPCR data and corresponding RNA-seq values obtained from biological replicates (Table 2) confirmed the reliability of the results (Pearson’s correlation, *r* > 0.92, *p* < 0.001).

### 3.2. SRT Interferes with T. rubrum Signaling and Regulation

Few kinase- and transcription factor-coding genes were modulated in response to SRT. The *T. rubrum* kinome (protein kinase superfamily members) is predicted to comprise 170 coding genes [32]. In our results, 81 kinases were modulated (Figure S1), coding chiefly for serine/threonine–protein kinases and kinases belonging to the CMGC (cyclin-dependent kinases (CDKs)) group. Among the 15 serine/threonine–protein kinase genes modulated, only TERG_07509 was repressed after 12 h of treatment. Among the eight CMGC-type kinases modulated by SRT exposure, TERG_12259 (CMGC/CLK protein kinase), TERG_05893 (CMGC protein kinase), and TERG_07061 (CMGC/SRPK protein kinase) were repressed. The latter two belong to the SRPK family and are active in the regulation of splicing by phosphorylation [33]. SRPKs act synergistically with CLK protein kinases, a family of Cdc2-like kinases that can auto-phosphorylate tyrosine residues, but phosphorylate their substrates exclusively on serine/threonine residues. The associated activity of SRPKs and CLK proteins phosphorylates SR (serine/arginine-rich) proteins, which mediate the phosphorylation of SR proteins and direct pre-mRNA splicing [34].

The comparative evaluation between anthropophilic and zoophilic dermatophytes revealed that only kinases and transcription factor Interpro (IPR) domain families were enriched in anthropophilic dermatophytes, suggesting that the modulation of signaling pathways and transcriptional regulation are key factors in host specificity [32]. Here, the reduced number of transcription factors modulated in response to SRT challenge (Figure S2) suggests the restrained infective potential of *T. rubrum* under SRT exposure. Most modulated transcription factors code for zinc finger domain-containing proteins and are preferentially repressed, mainly after 12 h of SRT exposure (including TERG_02532, TERG_01042, and TERG_05497), for which transcript accumulation increased over time.

### 3.3. Cell Wall and Membrane Structure Are Affected by SRT

SRT modulates the expression of genes associated with the formation of glucans and chitins, which are important structural polysaccharides in the fungal cell wall. These genes include endoglucanases (TERG_08178, TERG_03353, TERG_06189, and TERG_04268), chitinases (TERG_05626 and TERG_05625), and chitin synthases (TERG_12318 and TERG_12319). While glucan-related genes were predominantly induced after 12 h of treatment, chitin-associated genes were repressed after 12 h of SRT challenge. The hydrophobin-coding gene (TERG_04234) was repressed after only 12 h of SRT exposure.

**Figure 4:**
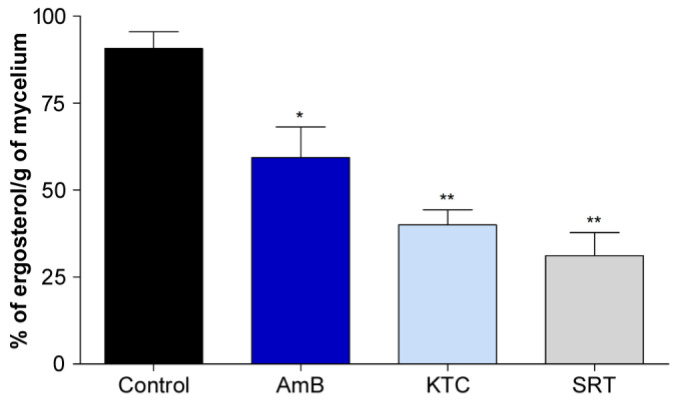
Effect of SRT on ergosterol content as a percentage of the wet weight of *T. rubrum.* Absence (negative control) and presence of amphotericin B (AMB, 1.25 µg/mL), ketoconazole (KTC, 4 µg/mL) (positive controls), and SRT (6.25 µg/mL). We tested the antifungals AMB and KTC at the minimal inhibitory concentration and the antidepressant SRT at a sub-inhibitory concentration. Asterisks represent statistical difference * *p* < 0.05 and ** *p* < 0.01 related to the control.

Furthermore, SRT was found to downregulate the expression of genes encoding components of the ergosterol biosynthetic pathway in *T. rubrum.* After 12 h, the genes that code for diphosphomevalonate decarboxylase (*erg*19; TERG_07616), C-8 sterol isomerase (*erg*1; TERG_06755), c-14 sterol reductase (*erg*24; TERG_04382), C-4 methylsterol oxidase (*erg*25; TERG_08545), ergosterol biosynthesis protein *erg*28 (TERG_04740), and sterol 24-C-methyltransferase/Delta (24(24[1]))-sterol reductase (*erg*4; TERG_06528/TERG_03102) were downregulated. The squalene epoxidase-coding gene (*erg*1; TERG_05717) was repressed after 3 and 12 h. The ergosterol dosage confirmed the inhibition of the biosynthetic pathway activity (Figure 4).

SRT, a cationic amphiphilic drug (CAD), causes drug-induced phospholipidosis (DIP) during excessive phospholipid storage within the lysosomes [18]. We observed the modulation of sphingomyelinase D (TERG_01406), lysosomal phospholipase A2 (TERG_03747), B (TERG_05522), and D (TERG_05303), and upregulation of secretory phospholipase A2 (TERG_00127).

Exposure to SRT led to the accumulation of transcripts of genes related to membrane transport. The number of modulated genes increased over time. Most downregulated genes at both time points are MFS transporters (3 of the 4 downregulated genes after 3 h, and 25 genes among the 38 downregulated genes after 12 h of SRT challenge). Among the upregulated genes, 23 remained upregulated after 3 and 12 h of drug challenge (Figure S3).

### 3.4. SRT Affects the Transcription of Oxidative Stress Genes in T. rubrum

**Figure 5:**
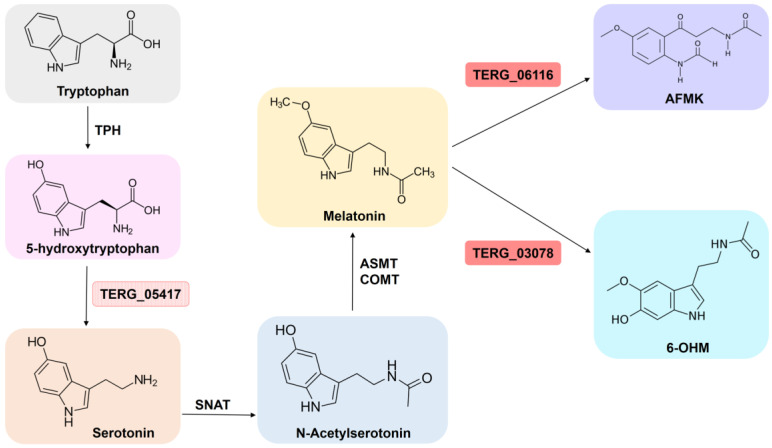
The biosynthetic pathway involved in synthesis of melatonin from tryptophan. Enzymatic steps from tryptophan to melatonin and the correlated metabolites. TPH: tryptophan hydroxylase; SNAT: serotonin N-acetyltransferase; ASMT: acetylserotonin O-methyltransferase; COMT: caffeic acid 3-O-methyltransferase; AFMK: N-acetyl-N-formyl-5-methoxykynuramine; 6-OHM: 6-hydroxymelatonin. The red genes are upregulated by *T. rubrum* in response to SRT. In pink, transcript accumulation is slightly below the log2 fold threshold (corresponding to 1.36 fold). Figure adapted from [36,37].

Through upregulating the glutathione S-transferase (GST) genes (TERG 03390, TERG 04960, TERG 01405, TERG 07326, TERG 02041, TERG 00579, TERG 08208, and TERG 05135), *T. rubrum* could have an altered oxidative stress response when exposed to SRT. These genes participate in the sequestration of endogenous or xenobiotic compounds [35]. In the presence of SRT, two other GST genes (TERG_06540 and TERG_06578), as well as genes encoding catalase A (TERG_01252) and Fe-superoxide dismutase (TERG_04819), were downregulated. Additionally, SRT upregulated the gene encoding thioredoxin reductase (TERG_08849) and downregulated the thioredoxin gene (TERG_05849). Thioredoxins (TRX) are small ubiquitous redox proteins that play key roles in redox signaling and oxidative stress responses. The genes responsible for the conversion of melatonin into two metabolites, 6-hydroxymelatonin and N-acetyl-N-formyl-5-methoxykynuramine, were also upregulated by SRT (Figure 5). Finally, the most induced gene at both time points (TERG_06548) was an oxidoreductase ortholog identified in *Microsporum canis.*

### 3.5. Effect of SRT on T. rubrum Metabolism

**Figure 6:**
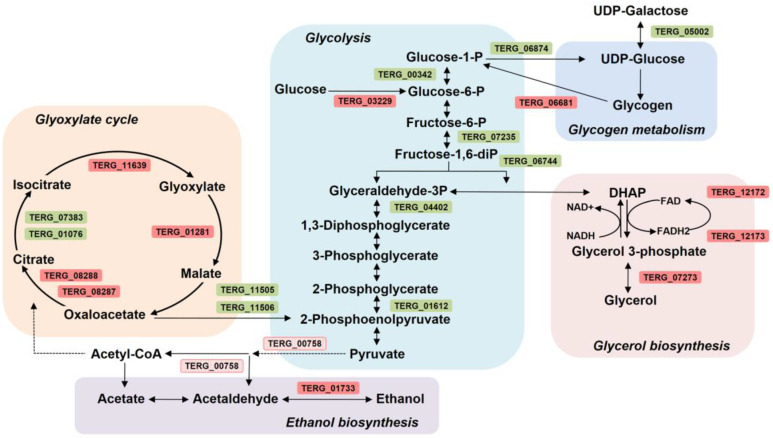
Schematic overview of the effect of SRT on the metabolism of *Trichophyton rubrum.* Genes induced (red) or repressed (green) considering log2 fold threshold ± 1.5. In pink, transcript accumulation is slightly below the log2 fold threshold (corresponding to 1.47 fold). UDP-galactose: uridine diphosphate galactose; UDP-glucose: uridine diphosphate glucose; DHAP: dihydroxyacetone phosphate.

SRT apparently interferes with the primary metabolism of *T. rubrum*. While genes involved in glycolysis are repressed, the genes involved in the glyoxylate cycle, ethanol and glycerol biosynthesis, and glycogen debranching are induced. The drug makes *T. rubrum* deviate from standard carbon metabolism to alternative pathways based on gene expression patterns (Figure 6). This repressive effect on glycolysis pathway genes counteracts the initial step performed by hexokinase (TERG_03229), which is induced. Metabolic reprogramming induced the expression of glyoxylate cycle-associated genes (TERG_11639, TERG_01281, TERG_08288, and TERG_08287) and glycerol biosynthesis genes (TERG_07273, TERG_12172, and TERG_12173). Additionally, SRT modulated ethanol- and glycerol-catabolism-related genes and induced a debranching enzyme (TERG_06681). This enzyme facilitates the breakdown of glycogen, which serves as a store of glucose.

## 4. Discussion

In addition to its use as an antidepressant, SRT has also been explored for other therapeutic applications. In vitro and in vivo studies have demonstrated its potential application in treating osteomyelitis infection [38] and cancer [39]. Furthermore, SRT presents antiparasitic [40], antibacterial [41], and antifungal activities against various pathogenic fungi, including *C. neoformans* [10,11], *Aspergillus fumigatus* [7], *Trichosporon asahii* [12], *Sporothrix schenckii* [9], and *T. rubrum* [15]. Through a drug repurposing approach, novel therapeutic options can rapidly enter clinical practice, as their safety and pharmacokinetics have been validated previously in patients. SRT is also effective alone or as an adjuvant therapeutic agent against fungal biofilm formation in vitro [15]. The synergistic effects of SRT combined with antifungals such as amphotericin B, caspofungin, or fluconazole have been documented in vitro [11,12,13,15]. Despite the potential therapeutic use of SRT in association with these drugs to control fungal infections, some are preferentially used intravenously.

### 4.1. SRT Affects Cell Signaling and Transcription in T. rubrum

Enrichment of the serine/threonine protein kinase domain (IPR002290), the catalytic domain of the protein kinase tyrosine, and the Zn(2)- C(6) fungal-like DNA-binding domain (IPR001138) has been reported in fungal anthropophilic organisms [32]. The transcriptional profile of *T. rubrum* after being challenged with SRT provided evidence that both kinases and transcriptional regulation-associated genes, when modulated, were preferentially induced, mainly at 12 h.

Phosphorylation by protein kinases is a critical mechanism that tunes cell activities, such as proliferation, metabolism, apoptosis, and gene expression. Cells possess thousands of kinases that phosphorylate specific sites in response to precise and controlled interactions [42]. The observed induced kinase modulation resulting from SRT exposure suggests the activation of specific signaling pathways. Our results indicate the activation of splicing events through the reversible phosphorylation of SR proteins. These phosphorylation events are dependent on both the SRPK and CLK families. The SRPK family of kinases phosphorylates SR proteins, promoting the nuclear import of SR proteins. Once in the nucleus, SR proteins may be further phosphorylated by the CLK family of kinases, which comprises a sequence of events essential for pre-mRNA splicing [33].

Conversely, the restricted number of protein kinase-coding genes modulated, considering the predicted whole kinome, implies the putative deactivation of other pathways. This deactivation strategy may represent a demand for energy conservation supported by, for example, the downregulation of ribosome biogenesis (Figure 2). Ribosome biogenesis is energetically expensive for cells. Consequently, unfavorable environmental conditions inhibit such processes [43].

Transcription factors are also associated with host specificity in dermatophytes [32]. We observed that a small number of transcriptional regulators were modulated, most of which had a zinc finger structural motif. Cells use transcription factors to dictate gene expression rates and regulate transcriptional networks [44]. Considering the relevance of transcription factor regulation and its key role in anthropophilic dermatophyte pathogenesis, we assume that drug challenge directly regulates transcription, possibly limiting energy expenditure under unfavorable conditions.

### 4.2. SRT Disturbs T. rubrum Cell Wall and Membrane

The fungal cell wall is a rigid layer with high plasticity that is essential for maintaining intracellular osmotic pressure [45]. β-1,3-glucan, chitin, and glycoproteins are primarily responsible for interactions between the cell and the environment. This wall is associated with a cell membrane that is predominantly composed of glycerophospholipids, sterols, and sphingolipids. Several molecules that form cell walls and membranes are exclusive to fungi and constitute suitable targets for antifungal development [45,46,47]. SRT exposure affects the expression of hydrolytic enzyme-coding genes in *T. rubrum*, which may contribute to changes in the cell wall structure.

Additionally, the repression of a beta-1,6 glucan synthetase that acts as a glycosidic linker suggests an imposed instability in cell wall assembly [48]. The overall repressive effects of SRT on the transcript levels for chitin synthases and chitinases may function as a compensatory mechanism to maintain cell wall integrity. This modulatory profile is similar to the effects of echinocandin antifungal agents on fungal cells. Echinocandins target the β-1,3-glucan synthase gene, deplete the glucan content of the cell wall, and increase chitin content.

Genes associated with ergosterol biosynthesis were downregulated after 12 h exposure to SRT, revealing that SRT affects plasma membrane properties. Moreover, SRT reduced ergosterol levels compared with amphotericin B and ketoconazole, which were used as positive controls (Figure 4). Amphotericin B complexes with ergosterol [49], whereas ketoconazole is a potent inhibitor of ergosterol biosynthesis [3]. Ergosterol modulates fluidity and permeability, interfering with water penetration [50]. The resultant alteration in the plasma membrane, together with the differential modulation of cell-wall-associated genes, suggests the occurrence of osmotic stress with alterations in the ergosterol composition of lipid bilayers, providing insights into the antifungal activity of SRT against *T. rubrum*.

Phospholipase A_2_ and sphingomyelinase D activities are downregulated after 12 h treatment with SRT. Lysosomal phospholipase A_2_ inhibition robustly correlates with drugs causing phospholipidosis [51], whereas the enzyme acid sphingomyelinase has been investigated as a potential target for antidepressant action [52]. Conversely, phospholipase B is upregulated at the initial SRT exposure, and phospholipase D is upregulated over time. Phospholipase B has hydrolase activity that cleaves fatty acids from phospholipids and lysophospholipids [53]. Phospholipase D comprises enzymes responsible for generating phosphatidic acid (PA), a putative secondary messenger implicated in the regulation of vesicular trafficking [54]. We observed that SRT also modulated genes involved in vesicular trafficking, including the induction of V-SNARE-coding genes (TERG_06294, TERG_07174) and the endoplasmic reticulum vesicle protein (TERG_00785) 25,388-fold induced after 3 h (log2 = 8.6). The secretory system of *T. rubrum* is essential for pathogenesis. Several keratinolytic enzymes are involved in the breakdown of keratin molecules, which may be assisted by ammonia and urea secretion [55]. The extra-vesicular content of *T. rubrum* is still not well understood. However, the vesicles of the dermatophyte *Trichophyton interdigitale* modulate the host immune response [56].

Besides affecting the cell wall structure and composition, global transcriptional analysis in response to SRT demonstrated the induction of drug resistance genes, mainly of the MFS genes, as a cellular stress response. The MFS is one of the largest groups of secondary active transporters and plays multiple roles in transporting a broad range of substrates, including sugars, amino acids, and drugs. The overall upregulated profile suggests a detoxification activity that increases with time since this family of proteins includes those encoding drug resistance transporters. However, at the 12 h time point, the downregulation of specific genes could represent an attempt to retain molecules such as phosphate (TERG_05891; MFS phosphate transporter) or monosaccharides (TERG_12194, TERG_04308; MFS sugar transporter), aiming to overcome the toxic drug effects.

### 4.3. SRT Overbalances Oxidative Stress Response

After 3 h of exposure to SRT, *T. rubrum* induced the expression of glutathione S-transferase genes to overcome its toxic effects. GSTs are small cytosolic proteins that contain a redox-active sulfhydryl group and are involved in cellular detoxification. Xenobiotics and endogenous products of oxidative stress are conjugated to glutathione (GSH) and secreted through vacuoles [57]. Endogenous toxic metabolites are removed through GSH-dependent detoxification processes. Thus, it confers protection against formaldehyde produced by methanol metabolism or against methylglyoxal, a byproduct of glycolysis [58,59]. Drug interactions with GSTs also facilitate their detoxification through GSH conjugation [60].

However, after 12 h of drug exposure, the downregulation of two glutathione transferase genes may reduce the efficiency of xenobiotic extrusion. Simultaneously, SRT downregulates Fe superoxide dismutase and catalase A genes, which neutralize reactive oxygen species (ROS). 

The genes encoding thioredoxin and thioredoxin reductase were oppositely modulated after 12 h of SRT exposure. The thioredoxin/thioredoxin reductase system (Trx/TrxR) confers redox homeostasis to fungal cells [61]. Because SRT downregulates TrxR, the control of the cellular redox balance remains compromised. In *A. fumigatus* and *C. neoformans*, the TrxR-encoding gene is essential for growth under in vitro conditions and shows weak homology to its human ortholog, making it a potential antifungal target [60,62].

*Saccharomyces cerevisiae* subjected to oxidative stress induced by H_2_O_2_ shows increased *TRX* transcription; melatonin, an antioxidant agent, partially mitigates oxidative stress by enhancing *TRX* mRNA accumulation in cells exposed to H_2_O_2_ [63]. Here, the upregulation of the genes encoding the enzymes responsible for the conversion of melatonin into its metabolites indicates a decrease in melatonin availability (Figure 5), followed by the upregulation of the *Trx* gene and the downregulation of *TrxR*. These data support the speculated dependency between *Trx* and melatonin availability, suggesting the counterbalanced expression of the thioredoxin/thioredoxin reductase system under experimental conditions.

In humans, SSRI drugs increase serotonin levels in synaptic spaces. Based on our data, SRT increased in *T. rubrum* transcripts of the gene encoding an aromatic-L-amino acid decarboxylase (EC 4.1.1.28) slightly below the log2 fold threshold (1.36 fold) (Figure 5). This enzyme catalyzes the conversion of 5-hydroxytryptophan to serotonin [36,37]. In *Aspergillus* spp., serotonin can directly kill fungi in vitro [64].

### 4.4. SRT Imposes Dynamic Metabolic Modulation

The overall profile showed flexibility in metabolic modulation in response to the toxic effect of SRT, activating alternative carbon metabolism. In general, microorganisms deplete preferred nutrients such as glucose by activating a regulatory cascade that represses the consumption of alternative carbon sources such as maltose, galactose, or ethanol [65]. The induction of a glycogen debranching enzyme and sugar transporters indicates the cellular effort to release glucose through glycogen degradation in response to energy deficiency. In our results based on analyses of transcript levels, although glucose is available (at least through glycogen metabolism) and the enzyme hexokinase is induced, activating the initial step of glycolysis that is responsible for the phosphorylation of glucose by ATP to glucose-6-P, the glycolytic fluxes are not directed downward toward the synthesis of two 2-phosphoenolpyruvate molecules. Carbon catabolite repression (CCR) is a mechanism that handles the utilization of an energy-efficient and readily available carbon source preferentially over a relatively less easily degradable carbon source. This mechanism helps microorganisms to obtain the maximum amount of glucose to provide carbon for biosynthetic processes [66]. We hypothesize that *T. rubrum* triggers a different strategy in response to SRT challenge, overcoming CCR and redirecting gene expression patterns, activating genes responsible for the catabolism of less favored carbon sources. A glucose reservoir may, somehow, counteract the harmful effects caused by SRT exposure. 

SRT treatment resulted in the repression of genes encoding glycolytic enzymes and an increase in the transcript levels of genes involved in alternative carbon utilization pathways, including the glyoxylate cycle. The induction of isocitrate lyase (TERG_11639) and malate synthase, glyoxysomal (TERG_01281) at the 3 h time point, and ATP-citrate synthases (TERG_08288/7) after 12 h drug exposure show the anabolic activity of the glyoxylate cycle counterbalancing glycolysis downregulation. The induction of the same isocitrate lyase (TERG_11639) at 48 and 96 h of *T. rubrum* incubation in keratin as the sole carbon source [55], coupled with the repression of genes related to glycolysis, suggests the promotion of a virulence response under both conditions. Glyoxylate cycle activation is directly associated with fungal pathogenicity [67,68]. Specifically, isocitrate lyase is a key enzyme in the glyoxylate cycle [69]. Our results suggest that the metabolic flexibility observed in *T. rubrum* in vitro presumably contributes to its fitness and pathogenicity in vivo.

Based on transcriptional profiling, we hypothesize that SRT induced ethanol metabolism via alcohol dehydrogenase activity, and glycerol metabolism via glycerol kinase modulation. Transcript accumulation of pyruvate decarboxylase (TERG_00758) slightly below the log2 fold threshold (corresponding to 1.47 fold) suggests the enzymatic catalysis of pyruvic acid to acetaldehyde and the subsequent ethanol production. Pyruvate decarboxylase catalyzes the decarboxylation of pyruvate to acetaldehyde with the release of carbon dioxide and is a key enzyme in ethanol fermentation [70]; thus, it directs the metabolic activity of *T. rubrum* to different alternative carbon pathways in response to SRT exposure.

## 5. Concluding Remarks, Challenges, and Perspectives

The limited number of antifungals available in the market and the increase in antifungal resistance highlight the urgent need for new antifungals with new targets. This approach requires detailed knowledge of the basic biology of the organism and intracellular processes essential for its growth and survival in the human host. Here, we assessed the transcriptional profile of the dermatophyte *T. rubrum*, focusing on the mechanisms activated to counteract the antifungal activity of SRT. The repositioning of SRT as an antifungal drug seems to be reliable from a molecular perspective. This drug targets important molecular events and metabolic pathways. With our results, it was possible to perceive the depth of the complexity of the regulatory systems that operate to restore damage but that can be useful to direct innovative strategies focusing on the weaknesses of dermatophytes, taking advantage of well-tolerated drugs for human use. SRT challenge analysis provided clues to understand the response and adaptive mechanisms of *T. rubrum* to drug exposure. Although its clinical use as an antifungal agent is promising, further studies are needed to validate our findings.

## Figures and Tables

**Table 1:** The most significantly up- or downregulated genes.

Upregulated		log_2_ (Fold Change)	
ID	Gene Product Name	3 h	12 h
TERG_06548	hypothetical protein (oxidoreductase, *M. canis*)	10.86	11.80
TERG_00785	endoplasmic reticulum vesicle protein 25	8.60	
TERG_01782	hypothetical protein	8.34	9.02
TERG_00010	amidase family protein (*T. verrucosum*)		8.72
TERG_03829	FAD binding domain-containing protein (*T. equinum*)		6.92
TERG_04952	multidrug resistance protein (*T. equinum*)	6.87	7.69
TERG_08954	hypothetical protein	6.61	
TERG_06106	sulfate permease 2 (*T. tonsurans*)		6.45
TERG_08751	ABC multidrug transporter, putative (*A. benhamiae*)	5.82	6.33
TERG_01543	S-adenosylmethionine (SAM)-dependent methyltransferase (*M. gypseum*)	5.42	7.48
TERG_08041	aminotransferase, putative (*A. benhamiae*)	5.42	
TERG_04937	alpha/beta hydrolase (*T. equinum*)	5.41	6.40
TERG_07830	hypothetical protein	5.41	8.22
**Downregulated**		**log_2_ (Fold Change)**	
**ID**	**Gene Product Name**	**3 h**	**12 h**
TERG_02653	hypothetical protein	−6.21	
TERG_02652	O-methyltransferase, putative (*T. verrucosum*)	−4.91	
TERG_04066	filamentation protein (Rhf1), putative (*T. verrucosum*)	−4.67	
TERG_02959	hypothetical protein	−4.16	
TERG_05816	hypothetical protein	−3.74	
TERG_02650	NmrA family protein (*T. equinum*)	−3.56	
TERG_12339	hypothetical protein	−3.31	
TERG_01619	toxin biosynthesis protein (Tri7), putative (*T. verrucosum*)	−3.20	
TERG_00490	erythromycin esterase (*T. tonsurans*)	−3.13	
TERG_03826	hypothetical protein	−2.77	
TERG_02959	hypothetical protein		−5.90
TERG_11536	hypothetical protein		−5.69
TERG_11771	hypothetical protein		−5.51
TERG_04742	hypothetical protein		−5.29
TERG_01148	hypothetical protein		−5.26
TERG_00490	erythromycin esterase (*T. tonsurans*)		−5.24
TERG_03919	phytoene dehydrogenase (*T. equinum*)		−5.20
TERG_01599	hypothetical protein		−4.95
TERG_12035	NB-ARC and TPR domain protein (*A. benhamiae*)		−4.87
TERG_11963	hypothetical protein		−4.79

**Table 2:** Comparison of the gene expression levels assayed by RNA sequencing and RT-qPCR.

ID	Condition	Gene Product Name	RNA-seq	RT-qPCR
TERG_11735	3 h	microtubule-associated protein (*T. tonsurans*)	−2.4	−1.41
TERG_07544	3 h	lipase (*T. tonsurans*)	2.02	1.13
TERG_06673	3 h	pachytene checkpoint component Pch2 (*T. tonsurans*)	2.0	0.70
TERG_01762	3 h	sulfite reductase (NADPH) hemoprotein beta-component	2.14	1.99
TERG_05522	3 h	lysophospholipase (*T. equinum*)	1.55	0.97
TERG_11924	12 h	ankyrin repeat protein (*T. tonsurans*)	3.85	1.06
TERG_06548	12 h	hypothetical protein (oxidoreductase, *M. canis*)	11.8	8.63
TERG_03936	12 h	CAMK protein kinase	−4.19	−0.09
TERG_06540	12 h	glutathione S-transferase (*T. tonsurans*)	−3.44	−0.07
TERG_07570	12 h	G-protein signaling regulator putative (*T. verrucosum*)	−3.47	−0.70
TERG_04234	12 h	hydrophobin putative (*T. verrucosum*)	−3.41	−1.98
TERG_07539	12 h	multidrug resistance protein (*T. tonsurans*)	−1.72	−1.61
TERG_03815	12 h	subtilisin-like protease 3	1.78	1.46

## Data Availability

We deposited RNA-sequencing (RNA-seq) data in the Gene Expression Omnibus (GEO) database under accession number GSE218521.

## Supplementary Materials

The following supporting information can be downloaded at: https://www.mdpi.com/article/10.3390/jof9020275/s1, Supplementary Figure S1. Kinase-related genes modulated in response to SRT. Supplementary Figure S2. Transcription-factor-related genes modulated in response to SRT. Supplementary Figure S3. *Trichophyton rubrum* membrane transporter genes modulated in response to SRT. Supplementary Table S1. List of primers used in RT-qPCR assays. Supplementary Table S2. General features of RNA-seq reads mapped to the *Trichophyton rubrum* reference genome. Supplementary Table S3. *Trichophyton rubrum* genes modulated in response to SRT exposure at each time point.
