# Supplementary material for: The Antidepressant Sertraline Affects Cell Signaling and Metabolism in Trichophyton rubrum
Source: J Fungi (Basel). 2023 Feb 20;9(2):275. doi: 10.3390/jof9020275 (PMC9961077; doi:10.3390/jof9020275)
Supplement: Supplementary file 1 [file jof-09-00275-s001.zip › jof-2136104-supplementary.pdf]

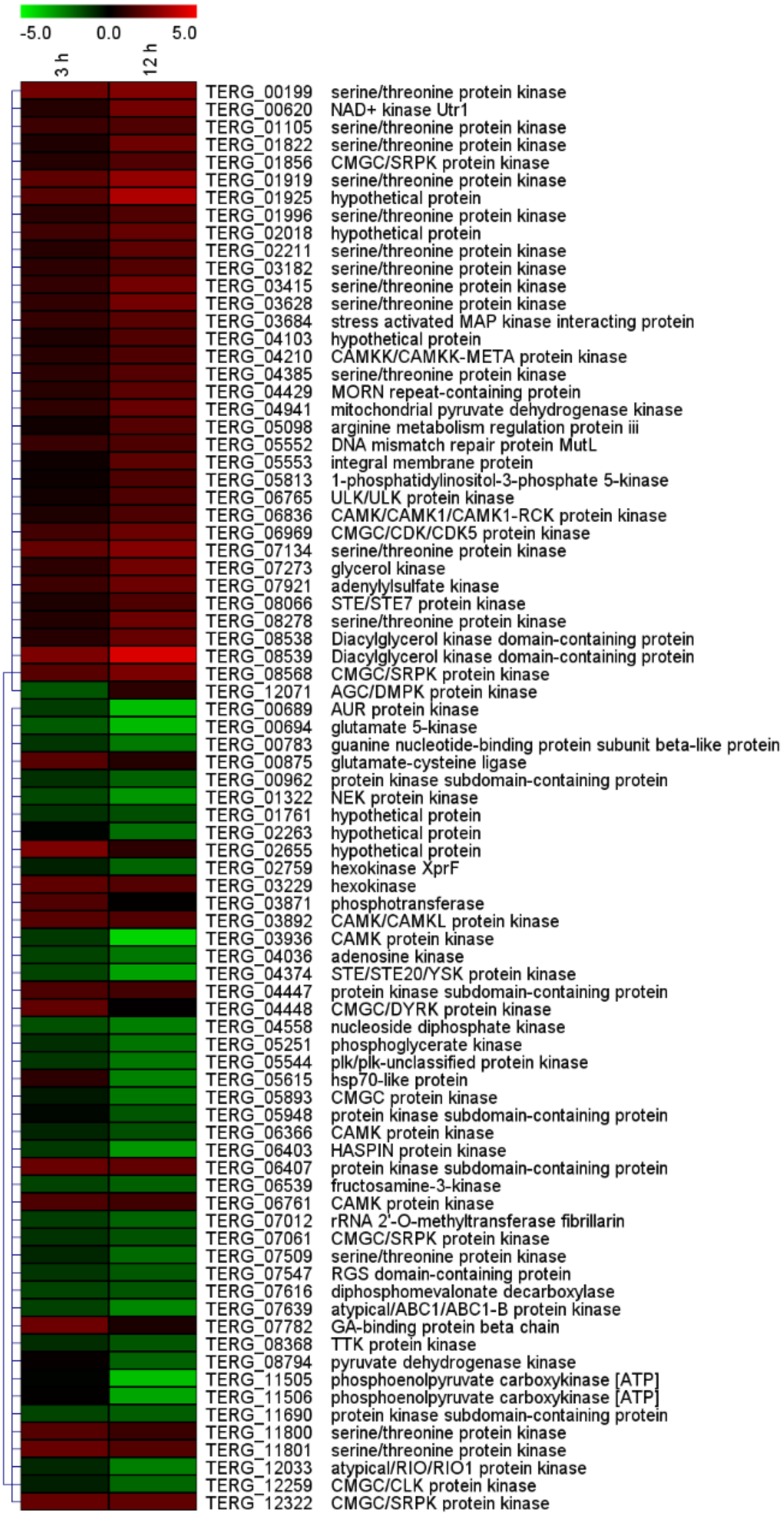

Figure S1: Kinase related genes modulated in response to SRT.

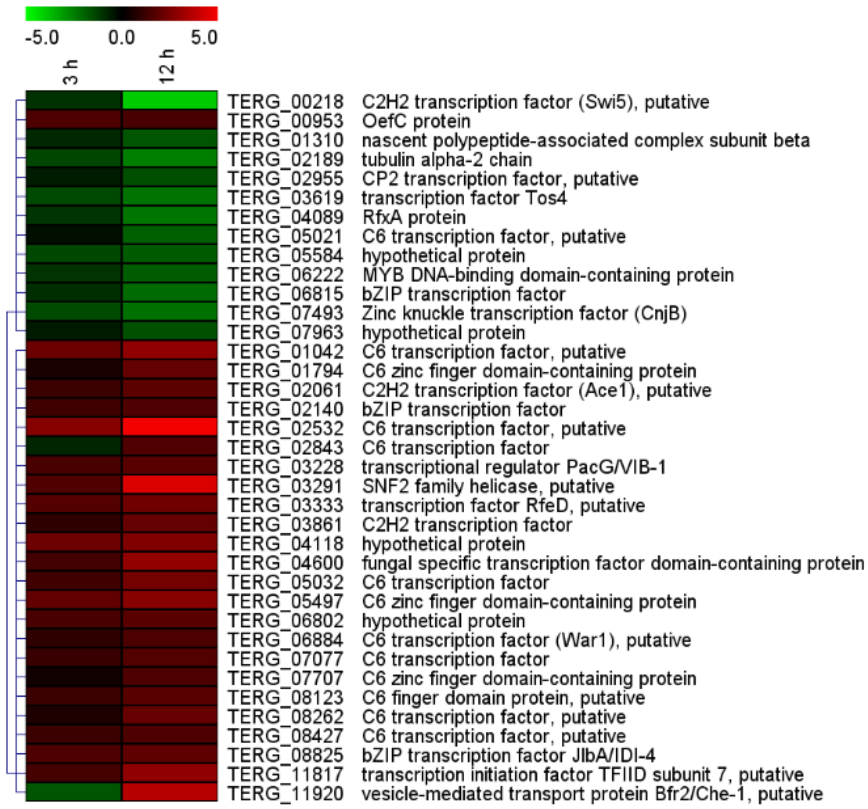

Figure S2: Transcription factors related genes modulated in response to SRT.

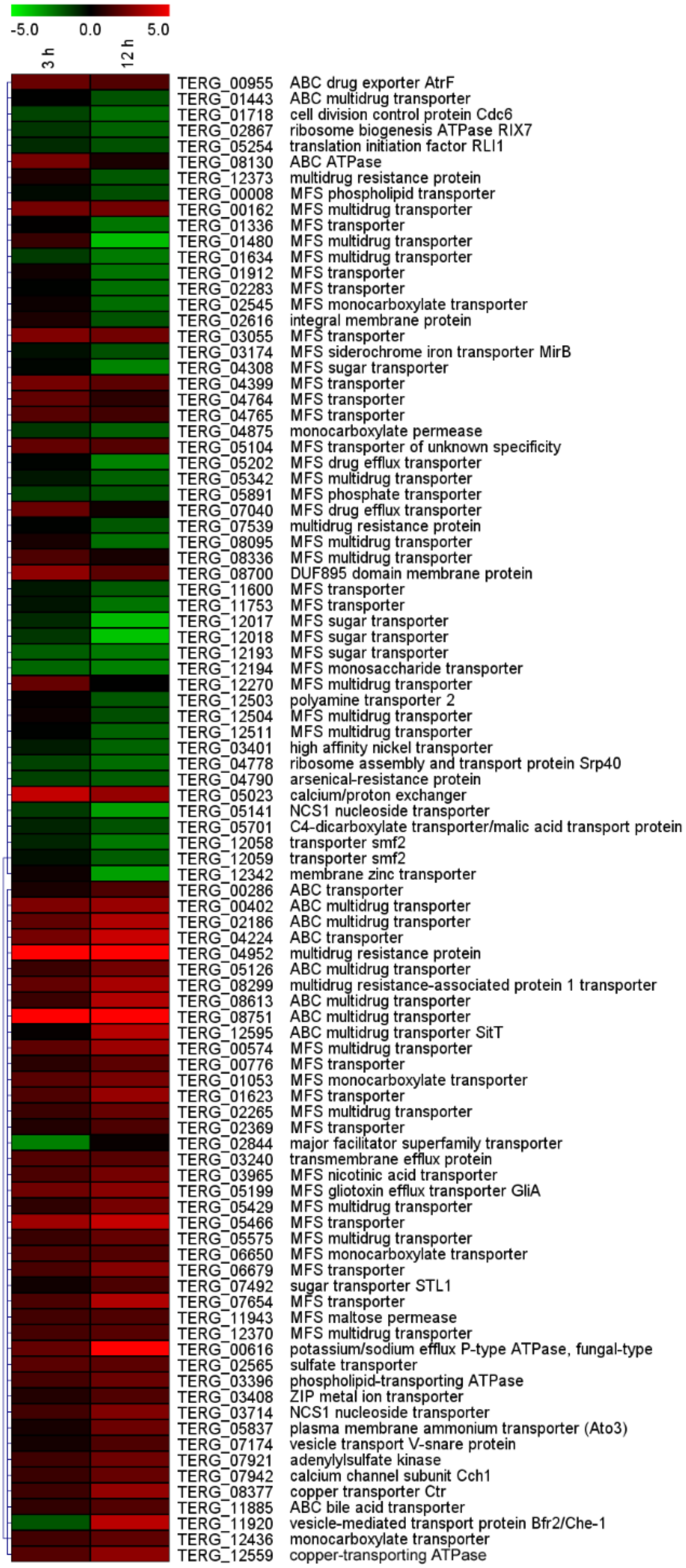

Figure S3: *Trichophyton rubrum* membrane transporter genes modulated in response to SRT.

Table S1: List of primers used in RT-qPCR assays.

ID	Gene Product Name	Primers (5' - 3')	Efficiency (%)
TERG_05742	DNA-dependent RNA polymerase II RPB140	FW: TGCAGGAGCTGGTGGAAGA<br>REV:GCTGGGAGGTACTGTTTGATCAA	94.99
TERG_04402	glyceraldehyde 3-phosphate dehydrogenase	FW: GCGTGACCCAGCCAACA<br>REV: CGGTGGACTCGACGATGTAGT	99.90
TERG_11735	microtubule associated protein (*T. tonsurans*)	FW: GGGCCAAAGAAACAACAACA<br>REV: CGCTCGGCATATTACCCTAA	98.2
TERG_07544	lipase (*T. tonsurans*)	FW: CTTGACGGATGGAAAAAGGA<br>REV: CACCGTTATTTGGATCAGCA	101.6
TERG_06673	pachytene checkpoint component Pch2 (*T. tonsurans*)	FW: ATGGGGAGATGCAGTTATGG<br>REV: ACAACGCTCACTTGTGCAAC	102.8
TERG_01762	sulfite reductase (NADPH) hemoprotein. beta-component	FW: ACGTGGATTCTGGGAAACAG<br>REV: AGGCCGAAAACGGAATACTT	95.3
TERG_05522	lysophospholipase (*T. equinum*)	FW: CAACGCGGACAATGTCTCTA<br>REV:CTCCATTCATCAAAGCACGA	104.0
TERG_11924	ankyrin repeat protein (*T. tonsurans*)	FW: GACCGGAAAGGTGGATGTTA<br>REV: CTCGTGTCGATAGCTTGCAG	100.0
TERG_06548	hypothetical protein	FW: ATCCGGTTCACAGACCAAAC<br>REV: GCTGGATTGAAAGGAGGACA	102.0
TERG_03936	CAMK protein kinase	FW: AGACAGCTGGCTTGCAAAAT<br>REV: TGAAACAGTCGTTTGGGACA	98.9
TERG_06540	glutathione transferase (*T. tonsurans*)	FW: AGGGATTGAACAAAGCCAGA<br>REV: AGGACCAATCTCATCCATCG	102.0
TERG_07570	G-protein signaling regulator. putative (*T. verrucosum*)	FW: TGGCATCAAGCACAAACTGT<br>REV: CTTTTTGGGCTTTGGACTTG	103.0
TERG_07539	multidrug resistance protein (*T. tonsurans*)	FW: CTCCTACCACCCCATTGCTA<br>REV: CTCAAGGCCGATAAGGAACA	100.0
TERG_03815	subtilisin-like protease 3	FW: TGGTGTTGACACCGGTATTG<br>REV: GTTGCCATCAGTGTTGTCGT	102.0

Table S2: General features of RNA-seq reads mapped to the *Trichophyton rubrum* reference genome.

Sample	Raw reads	High-quality reads	Mapped reads STAR	Total mapped reads (%)
Control 3 hours I	49,001,399	46,661,059	45,099,456	96.65
Control 3 hours II	53,711,149	50,929,711	48,747,310	95.71
Control 3 hours III	54,065,522	51,244,460	48,776,743	95.18
Control 12 hours I	49,723,884	47,231,185	45,011,034	95.30
Control 12 hours II	48,820,202	46,313,505	38,542,411	83.22
Control 12 hours III	49,378,560	46,733,086	38,063,153	81.45
SRT 3 hours I	44,238,209	41,936,258	40,012,345	95.41
SRT 3 hours II	46,989,637	44,653,620	41,137,174	92.13
SRT 3 hours III	46,798,013	44,244,584	42,078,809	95.10
SRT 12 hours I	42,267,590	40,220,692	38,407,036	95.49
SRT 12 hours II	43,358,547	41,011,155	38,955,931	94.99
SRT 12 hours III	36,247,455	34,371,291	32,688,609	95.10

Table S3: *Trichophyton rubrum* genes modulated in response to SRT exposure at each time point.

ID	3h	12h	Gene Product Name
TERG_00785	8.60		endoplasmic reticulum vesicle protein 25
TERG_02041	4.28		glutathione S-transferase (*T. equinum*)
TERG_12218	4.26		hypothetical protein
TERG_11932	3.77		hypothetical protein
TERG_11576	3.48		hypothetical protein
TERG_11896	3.47		aminotransferase (*T. equinum*)
TERG_06701	3.42		gamma-glutamyltransferase
TERG_12400	3.42		hypothetical protein
TERG_07781	3.36		hypothetical protein
TERG_00578	3.10		aspartic endopeptidase (AP1), putative (*T. verrucosum*)
TERG_05650	2.97		blasticidin-resistance protein (*T. tonsurans*)
TERG_04003	2.92		AMP dependent CoA ligase (*T. tonsurans*)
TERG_00911	2.85		hypothetical protein
TERG_02517	2.84		N-acetyltransferase, GNAT family, putative (*A. benhamiae*)
TERG_12606	2.78		protease DPPV, putative (*A. benhamiae*)
TERG_05943	2.73		aminotransferase, class III (*T. verrucosum*)
TERG_11700	2.69		ATP-dependent RNA helicase SUB2 (*T. equinum*)
TERG_06116	2.69		indoleamine 2,3-dioxygenase (*T. equinum*)
TERG_12661	2.60		hypothetical protein
TERG_00830	2.55		cytochrome P450 monooxygenase, putative (*A. benhamiae*)
TERG_04022	2.53		carboxypeptidase S1, putative (*A. benhamiae*)
TERG_01896	2.47		hypothetical protein
TERG_03070	2.47		flotillin domain-containing protein (*T. equinum*)
TERG_03489	2.46		hypothetical protein
TERG_02990	2.42		subtilisin-like protease 6
TERG_06781	2.40		metallo-beta-lactamase domain protein, putative (*A. benhamiae*)
TERG_05445	2.40		DUF323 domain-containing protein (*T. equinum*)
TERG_12173	2.36		glycerol-3-phosphate dehydrogenase, mitochondrial
TERG_08130	2.36		ABC ATPase (*T. equinum*)
TERG_12556	2.33		hypothetical protein
TERG_12135	2.33		hypothetical protein
TERG_08014	2.28		haloacid dehalogenase, type II
TERG_00227	2.28		glutamine amidotransferase subunit pdxT (*T. equinum*)
TERG_07451	2.27		acetoacetate-CoA ligase
TERG_01555	2.26		L-serine dehydratase, putative (*T. verrucosum*)
TERG_02615	2.24		oxidoreductase, 2OG-Fe(II) oxygenase family, putative (*A. benhamiae*)
TERG_01281	2.23		malate synthase, glyoxysomal
TERG_00579	2.23		glutathione S-transferase (*T. tonsurans*)
TERG_03490	2.22		hypothetical protein
TERG_07655	2.20		FAD dependent oxidoreductase, putative (*T. verrucosum*)
TERG_02360	2.19		hypothetical protein
TERG_01511	2.19		hypothetical protein
TERG_07782	2.19		GA-binding protein beta chain (*M. gypseum*)
TERG_08240	2.18		methyltransferase, putative (*T. verrucosum*)
TERG_11757	2.17		hypothetical protein
TERG_02803	2.15		3-oxoacyl-(acyl-carrier-protein) reductase (*T. verrucosum*)

TERG_01762	2.14	sulfite reductase (NADPH) hemoprotein, beta-component
TERG_08444	2.09	CipC protein (*M. canis*)
TERG_07980	2.09	hypothetical protein
TERG_05121	2.09	cytochrome P450 monooxygenase, putative (*A. benhamiae*)
TERG_04324	2.08	extracellular metalloproteinase 4
TERG_07040	2.07	MFS drug efflux transporter (*T. equinum*)
TERG_08757	2.06	hypothetical protein
TERG_07673	2.04	hypothetical protein
TERG_05347	2.03	epoxide hydrolase, putative (*T. verrucosum*)
TERG_08466	2.03	hypothetical protein
TERG_07001	2.03	hydroxyisocaproate dehydrogenase (*T. tonsurans*)
TERG_07544	2.02	lipase (*T. tonsurans*)
TERG_06673	2.00	pachytene checkpoint component Pch2 (*T. tonsurans*)
TERG_04448	1.97	CMGC/DYRK protein kinase (*M. gypseum*)
TERG_01463	1.97	cytochrome c peroxidase (*T. tonsurans*)
TERG_08151	1.96	GNAT family acetyltransferase, putative (*T. verrucosum*)
TERG_00614	1.96	dihydrodipicolinate synthetase (*T. tonsurans*)
TERG_07031	1.95	hypothetical protein
TERG_11539	1.95	3-oxoacyl-(acyl-carrier-protein) reductase (*T. tonsurans*)
TERG_00887	1.95	high expression lethality protein Hel10 (*T. tonsurans*)
TERG_04164	1.94	hypothetical protein
TERG_04764	1.93	MFS transporter (*T. tonsurans*)
TERG_06506	1.91	hypothetical protein
TERG_05677	1.91	phenazine biosynthesis protein (*T. equinum*)
TERG_12483	1.90	nonribosomal brevianamide peptide synthase FtmA (*T. verrucosum*)
TERG_00563	1.90	GNAT family N-acetyltransferase, putative (*A. benhamiae*)
TERG_04098	1.88	Swi5 domain-containing protein (*T. equinum*)
TERG_03855	1.88	WW domain-containing protein (*M. canis*)
TERG_02196	1.87	l-allo-threonine aldolase (*T. tonsurans*)
TERG_04021	1.87	GNAT family acetyltransferase, putative (*A. benhamiae*)
TERG_01270	1.87	AMP dependent ligase (*T. tonsurans*)
TERG_00413	1.86	DSBA oxidoreductase (*T. equinum*)
TERG_03886	1.84	proline permease, putative (*T. verrucosum*)
TERG_06806	1.82	hypothetical protein
TERG_12285	1.82	hypothetical protein
TERG_12581	1.82	hypothetical protein
TERG_07911	1.82	hypothetical protein
TERG_05357	1.81	succinate:fumarate antiporter (*T. tonsurans*)
TERG_01937	1.80	short chain dehydrogenase (*T. tonsurans*)
TERG_07327	1.80	pyridoxamine phosphate oxidase (*T. tonsurans*)
TERG_01445	1.79	hypothetical protein
TERG_11776	1.78	hypothetical protein
TERG_01088	1.78	3,4-dihydroxy-2-butanone-4-phosphate synthase
TERG_07734	1.78	O-methyltransferase, putative (*T. verrucosum*)
TERG_05641	1.78	amino acid permease (Can1), putative (*T. verrucosum*)
TERG_00875	1.76	glutamate-cysteine ligase
TERG_05077	1.76	D-amino-acid oxidase (*T. equinum*)
TERG_05528	1.76	hypothetical protein
TERG_07267	1.75	hypothetical protein

TERG_05545	1.75	GNAT family acetyltransferase, putative (*A. benhamiae*)
TERG_03791	1.74	NADH-ubiquinone oxidoreductase 20 kDa subunit (*T. equinum*)
TERG_11800	1.74	serine/threonine protein kinase (*T. equinum*)
TERG_01805	1.73	mucin (*M. canis*)
TERG_05484	1.73	acyl-CoA dehydrogenase (*T. tonsurans*)
TERG_04124	1.73	hypothetical protein
TERG_12248	1.72	transcription factor fet5 (*M. gypseum*)
TERG_00535	1.72	calcium-translocating P-type ATPase, PMCA-type
TERG_02837	1.71	3-beta hydroxysteroid dehydrogenase/isomerase family protein, putative (*T. verrucosum*)
TERG_00073	1.71	NADH-dependent flavin oxidoreductase, putative (*T. verrucosum*)
TERG_12005	1.71	hypothetical protein
TERG_12647	1.70	hypothetical protein
TERG_04765	1.70	MFS transporter, putative (*A. benhamiae*)
TERG_08614	1.69	hypothetical protein
TERG_06206	1.69	peptide-methionine (S)-S-oxide reductase
TERG_01330	1.66	GPI anchored protein, putative (*A. benhamiae*)
TERG_11538	1.66	3-oxoacyl-(acyl-carrier-protein) reductase (*T. tonsurans*)
TERG_08058	1.66	alpha-1,2-mannosidase family protein (*T. verrucosum*)
TERG_12250	1.66	hypothetical protein
TERG_01949	1.65	hypothetical protein
TERG_12295	1.65	hypothetical protein
TERG_01364	1.65	hypothetical protein
TERG_03893	1.64	C6 transcription factor, putative (*A. benhamiae*)
TERG_02214	1.64	carboxypeptidase 2
TERG_02249	1.63	phenylacetyl-CoA ligase (*T. tonsurans*)
TERG_02746	1.63	hypothetical protein
TERG_08405	1.63	leucine aminopeptidase 2
TERG_01429	1.63	hypothetical protein
TERG_05259	1.62	cytochrome P450, putative (*A. benhamiae*)
TERG_01733	1.60	alcohol dehydrogenase, putative (*T. verrucosum*)
TERG_08045	1.60	DUF455 domain-containing protein (*T. equinum*)
TERG_01436	1.60	oxidoreductase (*T. equinum*)
TERG_02798	1.59	DUF895 domain membrane protein (*T. equinum*)
TERG_01652	1.59	Atg10p (*M. gypseum*)
TERG_00953	1.58	OefC protein (*T. equinum*)
TERG_05305	1.58	glutathione-dependent formaldehyde-activating enzyme (*T. tonsurans*)
TERG_05714	1.57	hypothetical protein
TERG_05735	1.57	dipeptidyl peptidase 4
TERG_07282	1.56	cytochrome P450 monooxygenase, putative (*T. verrucosum*)
TERG_02179	1.56	keylime pathogenicity protein (*M. canis*)
TERG_08438	1.56	hypothetical protein
TERG_00070	1.56	maleylacetoacetate isomerase
TERG_08222	1.55	hypothetical protein
TERG_05522	1.55	lysophospholipase (*T. equinum*)
TERG_05409	1.55	FAD dependent oxidoreductase (*T. equinum*)
TERG_03422	1.54	hypothetical protein
TERG_04447	1.54	protein kinase subdomain-containing protein (*T. equinum*)
TERG_04948	1.53	hypothetical protein

TERG_02773	1.53	tartrate dehydrogenase
TERG_02725	1.53	hypothetical protein
TERG_08336	1.53	MFS multidrug transporter, putative (*A. benhamiae*)
TERG_06296	1.53	hypothetical protein
TERG_06761	1.53	CAMK protein kinase (*T. tonsurans*)
TERG_06047	1.52	hypothetical protein
TERG_08208	1.52	glutathione S-transferase (*T. verrucosum*)
TERG_00506	1.52	hypothetical protein
TERG_11639	1.51	isocitrate lyase
TERG_01732	1.51	amidohydrolase (*T. equinum*)
TERG_00487	1.50	hypothetical protein
TERG_12454	1.50	cutinase, putative (*A. benhamiae*)
TERG_08528	-1.51	fatty acid synthase beta subunit dehydratase (*T. tonsurans*)
TERG_00129	-1.56	hypothetical protein
TERG_08633	-1.59	hypothetical protein
TERG_05965	-1.60	hypothetical protein
TERG_08038	-1.83	polysaccharide deacetylase family protein (*T. equinum*)
TERG_07598	-1.84	serine carboxypeptidase (*T. equinum*)
TERG_02646	-1.96	hypothetical protein
TERG_02952	-2.05	oxidoreductase, short chain dehydrogenase/reductase family (*A. benhamiae*)
TERG_12514	-2.40	hypothetical protein
TERG_01347	-2.42	TIGR01456 family HAD hydrolase
TERG_11735	-2.50	microtubule associated protein (*T. tonsurans*)
TERG_02650	-3.56	NmrA family protein (*T. equinum*)
TERG_04066	-4.67	filamentation protein (Rhf1), putative (*T. verrucosum*)
TERG_02652	-4.91	O-methyltransferase, putative (*T. verrucosum*)

Gene expression values are expressed in $\log_2$ fold change.

ID	3h	12h	Gene Product Name
TERG_07028		6.08	hypothetical protein
TERG_12356		4.89	hypothetical protein
TERG_04373		4.52	hypothetical protein
TERG_06992		4.36	pyridine nucleotide-disulphide oxidoreductase (*T. tonsurans*)
TERG_03254		4.22	hypothetical protein
TERG_01717		4.13	RTA1 domain protein, putative (*A. benhamiae*)
TERG_12014		3.99	hypothetical protein
TERG_06772		3.88	hypothetical protein
TERG_03252		3.85	hypothetical protein
TERG_11924		3.85	ankyrin repeat protein (*T. tonsurans*)
TERG_03293		3.83	hypothetical protein
TERG_12197		3.72	tyrosyl-DNA phosphodiesterase (*T. tonsurans*)
TERG_11920		3.68	vesicle-mediated transport protein Bfr2/Che-1, putative (*T. verrucosum*)
TERG_05823		3.67	integral membrane protein (*A. benhamiae*)
TERG_06769		3.64	hypothetical protein
TERG_06839		3.60	extracellular proline-rich protein (*T. verrucosum*)
TERG_04183		3.59	yqcI protein (*T. equinum*)
TERG_08613		3.50	ABC multidrug transporter, putative (*A. benhamiae*)

TERG_07654	3.48	MFS transporter, putative (*T. verrucosum*)
TERG_01154	3.40	RTA1 domain protein, putative (*T. verrucosum*)
TERG_03712	3.39	hydantoinase (*T. equinum*)
TERG_08049	3.38	cation diffusion facilitator, putative (*T. verrucosum*)
TERG_03552	3.36	serine carboxypeptidase (*T. tonsurans*)
TERG_05428	3.34	hypothetical protein
TERG_08508	3.28	hypothetical protein
TERG_01394	3.27	cytochrome P450 alkane hydroxylase, putative (*T. verrucosum*)
TERG_03251	3.19	hypothetical protein
TERG_02830	3.19	hypothetical protein
TERG_06167	3.16	hypothetical protein
TERG_07133	3.16	hypothetical protein
TERG_06770	3.16	cytochrome P450, putative (*T. verrucosum*)
TERG_00801	3.11	hypothetical protein
TERG_06189	3.04	endo-1,3(4)-beta-glucanase, putative (*T. verrucosum*)
TERG_08347	2.99	cytochrome P450 monooxygenase, putative (*A. benhamiae*)
TERG_04600	2.96	fungal specific transcription factor domain-containing protein (*T. equinum*)
TERG_12118	2.96	hypothetical protein
TERG_08801	2.94	hypothetical protein
TERG_08377	2.92	copper transporter Ctr (*T. tonsurans*)
TERG_11817	2.91	transcription initiation factor TFIID subunit 7, putative (*T. verrucosum*)
TERG_01678	2.90	histone-lysine N-methyltransferase (*T. tonsurans*)
TERG_04714	2.89	mitochondrial phosphate carrier protein (*T. tonsurans*)
TERG_00917	2.89	aureobasidin A resistance protein (*T. equinum*)
TERG_02306	2.86	integral membrane protein (*T. equinum*)
TERG_03491	2.81	hypothetical protein
TERG_11513	2.79	esterase (*T. equinum*)
TERG_11774	2.77	hypothetical protein
TERG_12718	2.77	hypothetical protein
TERG_04109	2.74	C3HC4 finger protein (*A. benhamiae*)
TERG_00149	2.72	hypothetical protein
TERG_12070	2.72	hypothetical protein
TERG_03183	2.71	hypothetical protein
TERG_01970	2.70	hypothetical protein
TERG_02150	2.67	hypothetical protein
TERG_06679	2.64	MFS transporter, putative (*A. benhamiae*)
TERG_12068	2.63	PLC-E (*T. equinum*)
TERG_12237	2.63	hypothetical protein
TERG_12679	2.62	JmjC domain-containing protein (*T. tonsurans*)
TERG_05618	2.61	Lcc2 (*T. equinum*)
TERG_02213	2.60	extracellular metalloproteinase 1
TERG_07919	2.59	thioesterase family protein (*T. verrucosum*)
TERG_07982	2.59	hypothetical protein
TERG_08855	2.58	hypothetical protein
TERG_03868	2.56	cupin domain-containing protein (*T. tonsurans*)
TERG_00527	2.55	hypothetical protein
TERG_03714	2.53	NCS1 nucleoside transporter (*T. tonsurans*)
TERG_12028	2.53	metallothionein family protein (*T. equinum*)
TERG_05298	2.53	rhomboid family membrane protein (*T. tonsurans*)

TERG_04721	2.52	glutamate carboxypeptidase (*T. equinum*)
TERG_01694	2.51	ankyrin repeat-containing protein (*T. equinum*)
TERG_01807	2.51	hypothetical protein
TERG_11537	2.49	hypothetical protein
TERG_05988	2.49	solid-state culture expressed protein (Aos23), putative (*T. verrucosum*)
TERG_04939	2.47	hypothetical protein
TERG_11514	2.47	lipase/esterase, putative (*T. verrucosum*)
TERG_07331	2.45	integral membrane protein (*T. tonsurans*)
TERG_12522	2.45	4-hydroxyphenylpyruvate dioxygenase (*T. tonsurans*)
TERG_06290	2.44	hypothetical protein
TERG_08230	2.44	hypothetical protein
TERG_03132	2.43	hypothetical protein
TERG_03830	2.43	hypothetical protein
TERG_07479	2.41	hypothetical protein
TERG_01225	2.40	GTPase-activating protein GYP7 (*T. equinum*)
TERG_11517	2.40	hypothetical protein
TERG_12639	2.39	choriogenin Hminor (*M. canis*)
TERG_06807	2.39	cutinase, putative (*A. benhamiae*)
TERG_02562	2.38	chitin synthase C
TERG_07242	2.37	di-trans,poly-cis-decaprenylcistransferase
TERG_03722	2.37	hypothetical protein
TERG_02448	2.36	hypothetical protein
TERG_05032	2.36	C6 transcription factor (*T. equinum*)
TERG_01265	2.36	HhH-GPD family base excision DNA repair protein (*T. tonsurans*)
TERG_03329	2.36	hypothetical protein
TERG_03218	2.35	extracellular salicylate hydroxylase/monooxygenase, putative (*T. verrucosum*)
TERG_03732	2.35	hypothetical protein
TERG_03570	2.34	mitochondria fission 1 protein
TERG_00638	2.34	cellobiose dehydrogenase, putative (*A. benhamiae*)
TERG_05429	2.33	MFS multidrug transporter, putative (*T. verrucosum*)
TERG_03415	2.33	serine/threonine protein kinase
TERG_12692	2.33	hypothetical protein
TERG_02757	2.33	NADP-dependent alcohol dehydrogenase (*T. equinum*)
TERG_08922	2.32	hypothetical protein
TERG_05013	2.32	stress response RCI peptide (*T. equinum*)
TERG_03965	2.31	MFS nicotinic acid transporter (*T. tonsurans*)
TERG_12337	2.30	hypothetical protein
TERG_03628	2.30	serine/threonine protein kinase (*T. tonsurans*)
TERG_07705	2.30	hypothetical protein
TERG_00620	2.30	NAD+ kinase Utr1 (*T. equinum*)
TERG_00847	2.30	SOH1 family protein (*T. equinum*)
TERG_00959	2.30	RNA binding protein, putative (*A. benhamiae*)
TERG_05135	2.30	glutathione S-transferase GliG (*A. benhamiae*)
TERG_08686	2.29	hypothetical protein
TERG_00950	2.29	hypothetical protein
TERG_07273	2.29	glycerol kinase (*T. equinum*)
TERG_08622	2.28	transferase (*M. canis*)
TERG_06402	2.28	Ser/Thr protein phosphatase family protein (*T. verrucosum*)
TERG_05239	2.28	DNA polymerase POL4, putative (*T. verrucosum*)

TERG_07311	2.28	fibronectin type III domain-containing protein (*T. equinum*)
TERG_05126	2.27	ABC multidrug transporter, putative (*A. benhamiae*)
TERG_03511	2.27	calcineurin binding protein (*T. equinum*)
TERG_05310	2.27	kynurenine 3-monooxygenase (*T. tonsurans*)
TERG_07804	2.26	PBSP domain-containing protein (*T. tonsurans*)
TERG_12045	2.26	hypothetical protein
TERG_05244	2.26	hypothetical protein
TERG_05349	2.26	oxidoreductase, 2OG-Fe(II) oxygenase family, putative (*A. benhamiae*)
TERG_05680	2.26	IBR domain containing protein (*T. equinum*)
TERG_06900	2.25	hypothetical protein
TERG_00471	2.25	hypothetical protein
TERG_12694	2.24	hypothetical protein
TERG_01171	2.24	autophagy regulatory protein Atg2, putative (*T. verrucosum*)
TERG_03662	2.23	hypothetical protein
TERG_03620	2.23	glycosyl transferase (*T. tonsurans*)
TERG_11831	2.22	hypothetical protein
TERG_11543	2.22	hypothetical protein
TERG_01346	2.22	lipase/serine esterase (*T. tonsurans*)
TERG_06994	2.22	hypothetical protein
TERG_00416	2.22	phenylacetaldoxime dehydratase family protein, putative (*A. benhamiae*)
TERG_03512	2.21	hypothetical protein
TERG_07987	2.21	GNAT family acetyltransferase (*T. equinum*)
TERG_00410	2.21	hypothetical protein
TERG_01822	2.21	serine/threonine protein kinase
TERG_06766	2.20	high affinity methionine permease (*T. tonsurans*)
TERG_06995	2.19	metalloreductase, putative (*T. verrucosum*)
TERG_08278	2.19	serine/threonine protein kinase (*T. tonsurans*)
TERG_07501	2.19	nitrate reductase (*T. tonsurans*)
TERG_12243	2.18	hypothetical protein
TERG_02558	2.18	oxysterol binding protein (*T. tonsurans*)
TERG_07891	2.18	DUF255 domain-containing protein (*T. equinum*)
TERG_07921	2.18	adenylylsulfate kinase
TERG_03064	2.17	MCAK-like kinesin (*T. equinum*)
TERG_06969	2.17	CMGC/CDK/CDK5 protein kinase
TERG_12722	2.17	transferase (*M. canis*)
TERG_03177	2.17	RING finger domain protein, putative (*A. benhamiae*)
TERG_01849	2.17	hypothetical protein
TERG_02469	2.15	hypothetical protein
TERG_11680	2.15	DEAD/DEAH box DNA helicase (*T. tonsurans*)
TERG_08065	2.14	auxin Efflux Carrier superfamily protein (*T. equinum*)
TERG_03763	2.14	AAA family ATPase (*T. tonsurans*)
TERG_05837	2.12	plasma membrane ammonium transporter (Ato3), putative (*A. benhamiae*)
TERG_05541	2.12	hypothetical protein
TERG_02340	2.11	polysaccharide deacetylase (*T. equinum*)
TERG_06741	2.11	ubiquitin C-terminal hydrolase (*T. tonsurans*)
TERG_03790	2.11	DUF1275 domain-containing protein (*T. equinum*)
TERG_03396	2.11	phospholipid-transporting ATPase (*T. equinum*)
TERG_04473	2.11	hypothetical protein
TERG_03367	2.11	hypothetical protein

TERG_03443	2.10	ankyrin repeat protein (*T. equinum*)
TERG_04698	2.10	hypothetical protein
TERG_12711	2.10	hypothetical protein
TERG_01626	2.09	AT hook domain-containing protein family protein (*T. equinum*)
TERG_11681	2.09	DEAD/DEAH box DNA helicase (*T. tonsurans*)
TERG_02092	2.09	hypothetical protein
TERG_11764	2.09	proline-threonine-rich repeat protein (*T. tonsurans*)
TERG_02609	2.08	actin patches distal protein 1 (*T. equinum*)
TERG_12721	2.08	transferase (*M. canis*)
TERG_05706	2.08	hypothetical protein
TERG_04941	2.08	mitochondrial pyruvate dehydrogenase kinase (*T. tonsurans*)
TERG_12455	2.08	hypothetical protein
TERG_04283	2.07	hypothetical protein
TERG_03537	2.07	alpha/beta hydrolase (*T. equinum*)
TERG_07252	2.07	hypothetical protein
TERG_05736	2.07	alkaline serine protease (*T. tonsurans*)
TERG_08301	2.07	fatty-acid synthase subunit alpha (*M. canis*)
TERG_00273	2.07	mucin (*M. canis*)
TERG_00188	2.06	hypothetical protein
TERG_08538	2.06	Diacylglycerol kinase domain-containing protein (*T. equinum*)
TERG_08262	2.06	C6 transcription factor, putative (*A. benhamiae*)
TERG_02651	2.05	NRPS-like enzyme, putative (*A. benhamiae*)
TERG_11941	2.05	MCAK-like kinesin (*T. equinum*)
TERG_08666	2.05	hypothetical protein
TERG_05111	2.05	hypothetical protein
TERG_03125	2.05	hypothetical protein
TERG_02113	2.04	TIGR01456 family HAD hydrolase
TERG_01794	2.04	C6 zinc finger domain-containing protein (*M. canis*)
TERG_03681	2.04	leucine aminopeptidase (*T. equinum*)
TERG_07779	2.03	LCCL domain-containing protein (*T. tonsurans*)
TERG_05876	2.03	hypothetical protein
TERG_03861	2.03	C2H2 transcription factor (*T. tonsurans*)
TERG_02018	2.02	hypothetical protein
TERG_05425	2.02	guanine deaminase
TERG_02315	2.02	extracellular aspartic endopeptidase, putative (*A. benhamiae*)
TERG_05441	2.02	cytochrome P450 alkane hydroxylase, putative (*T. verrucosum*)
TERG_05157	2.02	hypothetical protein
TERG_02265	2.02	MFS multidrug transporter (*T. equinum*)
TERG_08384	2.02	hypothetical protein
TERG_06145	2.02	arrestin (*T. equinum*)
TERG_12615	2.01	ankyrin repeat-containing protein (*T. equinum*)
TERG_06735	2.01	oxysterol binding protein (*T. tonsurans*)
TERG_06778	2.01	PAP2 domain protein (*T. verrucosum*)
TERG_00362	2.01	glutaminase (*T. equinum*)
TERG_07942	2.01	calcium channel subunit Cch1 (*T. tonsurans*)
TERG_07990	2.00	proline-rich protein (*T. tonsurans*)
TERG_02974	2.00	hypothetical protein
TERG_06457	2.00	hypothetical protein
TERG_12588	2.00	spindle pole body associated protein SnaD, putative (*T. verrucosum*)

TERG_03061	2.00	hypothetical protein
TERG_04268	2.00	cell wall glucanase, putative (*A. benhamiae*)
TERG_02483	1.99	hypothetical protein
TERG_08317	1.99	hypothetical protein
TERG_02371	1.99	hypothetical protein
TERG_05422	1.98	hypothetical protein
TERG_02298	1.98	GPI anchored cell wall protein, putative (*T. verrucosum*)
TERG_02902	1.98	hypothetical protein
TERG_05575	1.98	MFS multidrug transporter (*T. tonsurans*)
TERG_06955	1.97	hypothetical protein
TERG_03854	1.97	laccase (*T. tonsurans*)
TERG_07983	1.96	hypothetical protein
TERG_03121	1.96	DNA repair and recombination protein RAD26 (*M. canis*)
TERG_05563	1.95	hypothetical protein
TERG_03857	1.95	phosphotransferase enzyme family protein (*T. equinum*)
TERG_05079	1.95	extracellular OTU-like cysteine protease, putative (*A. benhamiae*)
TERG_03848	1.95	hypothetical protein
TERG_12072	1.93	hypothetical protein
TERG_03969	1.93	hypothetical protein
TERG_04376	1.93	aldehyde reductase I (ARI), putative (*T. verrucosum*)
TERG_07871	1.93	hypothetical protein
TERG_12616	1.92	ankyrin repeat-containing protein (*T. equinum*)
TERG_00995	1.92	hypothetical protein
TERG_04785	1.92	hypothetical protein
TERG_07870	1.92	glycosyl transferase, putative (*A. benhamiae*)
TERG_02183	1.92	C2H2 finger domain-containing protein (*T. equinum*)
TERG_02885	1.91	E3 ubiquitin-protein ligase pub1 (*T. tonsurans*)
TERG_00828	1.90	hypothetical protein
TERG_00233	1.90	glyoxalase (*T. tonsurans*)
TERG_05553	1.90	integral membrane protein (*T. equinum*)
TERG_05446	1.90	sarcosine oxidase (*T. equinum*)
TERG_08341	1.90	hypothetical protein
TERG_07991	1.89	proline-rich protein (*T. tonsurans*)
TERG_01695	1.89	hypothetical protein
TERG_02061	1.89	C2H2 transcription factor (Ace1), putative (*T. verrucosum*)
TERG_08328	1.89	basic proline-rich protein (*M. canis*)
TERG_00176	1.88	hydroxyisourate hydrolase
TERG_03549	1.88	hypothetical protein
TERG_12436	1.88	monocarboxylate transporter (*M. canis*)
TERG_02211	1.88	serine/threonine protein kinase (*T. tonsurans*)
TERG_00439	1.87	hypothetical protein
TERG_04417	1.87	DUF455 domain protein (*A. benhamiae*)
TERG_06771	1.87	FAD binding domain protein (*A. benhamiae*)
TERG_06368	1.87	hypothetical protein
TERG_06552	1.87	aspartic-type endopeptidase (OpsB), putative (*T. verrucosum*)
TERG_12139	1.86	hypothetical protein
TERG_02714	1.86	proteinase, putative (*A. benhamiae*)
TERG_01173	1.86	hypothetical protein
TERG_03179	1.85	NlpC/P60-like cell-wall peptidase, putative (*A. benhamiae*)

TERG_00059	1.84	hypothetical protein
TERG_04429	1.84	MORN repeat-containing protein (*T. equinum*)
TERG_05452	1.84	GABA permease (*T. tonsurans*)
TERG_05909	1.83	hypothetical protein
TERG_03562	1.83	hypothetical protein
TERG_06978	1.83	SirQ protein (*T. equinum*)
TERG_03945	1.83	hypothetical protein
TERG_03789	1.83	glutamine-serine rich protein MS8, putative (*A. benhamiae*)
TERG_11762	1.83	lysine-specific permease (*M. gypseum*)
TERG_08601	1.83	hypothetical protein
TERG_02453	1.83	hypothetical protein
TERG_00776	1.82	MFS transporter (*T. tonsurans*)
TERG_03684	1.82	stress activated MAP kinase interacting protein (*T. equinum*)
TERG_00590	1.82	hypothetical protein
TERG_03456	1.82	hypothetical protein
TERG_07265	1.82	sucrase/ferredoxin domain-containing protein (*T. equinum*)
TERG_00517	1.81	hypothetical protein
TERG_02561	1.81	ubiquinone biosynthesis protein Coq7 (*T. tonsurans*)
TERG_07143	1.81	potassium/sodium efflux P-type ATPase, fungal-type
TERG_04543	1.81	aminotransferase GliI (*T. equinum*)
TERG_08069	1.81	glutathione-disulfide reductase
TERG_03486	1.80	Lectin C-type domain protein (*T. verrucosum*)
TERG_02412	1.80	HHE domain protein (*T. verrucosum*)
TERG_00252	1.80	hypothetical protein
TERG_06044	1.80	hypothetical protein
TERG_03228	1.80	transcriptional regulator PacG/VIB-1 (*T. verrucosum*)
TERG_08123	1.80	C6 finger domain protein, putative (*A. benhamiae*)
TERG_04774	1.80	isoflavone reductase family protein (*T. verrucosum*)
TERG_12349	1.80	hypothetical protein
TERG_12114	1.79	hypothetical protein
TERG_06460	1.79	LEM3/CDC50 family protein (*T. tonsurans*)
TERG_05304	1.79	cyanate hydratase
TERG_03653	1.79	hypothetical protein
TERG_12075	1.79	hypothetical protein
TERG_06802	1.79	hypothetical protein
TERG_07188	1.79	xanthine dehydrogenase (*T. tonsurans*)
TERG_06594	1.78	DUF1183 domain-containing protein (*T. equinum*)
TERG_12176	1.78	hypothetical protein
TERG_11634	1.77	DUF907 domain protein (*T. verrucosum*)
TERG_00331	1.77	golgi apparatus membrane protein TVP18 (*T. tonsurans*)
TERG_02632	1.77	autophagy cysteine endopeptidase Atg4, putative (*A. benhamiae*)
TERG_01049	1.77	hypothetical protein
TERG_06801	1.77	cyclic nucleotide-binding domain-containing protein (*T. tonsurans*)
TERG_12055	1.77	vegetative cell wall protein gp1 (*M. canis*)
TERG_02063	1.76	hypothetical protein
TERG_04915	1.76	alpha/beta hydrolase (*T. tonsurans*)
TERG_08100	1.76	CDP-diacylglycerol-inositol 3-phosphatidyltransferase PIS (*T. equinum*)
TERG_12712	1.76	hypothetical protein
TERG_00483	1.76	hypothetical protein

TERG_04338	1.75	subtilisin-like serine protease pepC
TERG_05136	1.75	cytochrome P450 oxidoreductase GliC (*A. benhamiae*)
TERG_12265	1.75	glutathione-dependent formaldehyde-activating enzyme (*T. tonsurans*)
TERG_01546	1.75	oxidoreductase (*T. tonsurans*)
TERG_07489	1.75	hypothetical protein
TERG_06543	1.75	phosphatidate cytidylyltransferase (*T. tonsurans*)
TERG_12516	1.75	DNA binding protein SART-1 (*T. tonsurans*)
TERG_01551	1.75	hypothetical protein
TERG_08255	1.75	carboxypeptidase S1, putative (*A. benhamiae*)
TERG_06239	1.75	hypothetical protein
TERG_01328	1.74	metalloreductase transmembrane component, putative (*A. benhamiae*)
TERG_04385	1.74	serine/threonine protein kinase (*T. tonsurans*)
TERG_08535	1.74	RING finger protein (*A. benhamiae*)
TERG_05098	1.74	arginine metabolism regulation protein iii (*T. verrucosum*)
TERG_11795	1.74	hypothetical protein
TERG_01889	1.74	RING finger domain-containing protein (*T. tonsurans*)
TERG_06089	1.74	PH domain-containing protein (*T. equinum*)
TERG_00338	1.74	glutamate carboxypeptidase (*T. tonsurans*)
TERG_03705	1.74	cytochrome P450 monooxygenase, putative (*A. benhamiae*)
TERG_00148	1.74	hypothetical protein
TERG_02918	1.74	alkaline phytoceramidase (*T. equinum*)
TERG_00100	1.73	flavin-containing monooxygenase (*T. equinum*)
TERG_02222	1.73	TBC domain-containing protein (*T. equinum*)
TERG_05842	1.73	png1 (*T. equinum*)
TERG_00836	1.73	QDE-2-interacting protein (*M. gypseum*)
TERG_00954	1.72	hypothetical protein
TERG_03283	1.72	dienelactone hydrolase (*M. gypseum*)
TERG_07666	1.72	hypothetical protein
TERG_12370	1.72	MFS multidrug transporter, putative (*A. benhamiae*)
TERG_06354	1.72	SVP1-like protein (*T. tonsurans*)
TERG_07762	1.71	hypothetical protein
TERG_00680	1.71	fimbrin (*T. tonsurans*)
TERG_11557	1.71	luciferin 4-monooxygenase (*T. equinum*)
TERG_01644	1.71	SNF2 family helicase/ATPase (*T. equinum*)
TERG_01357	1.71	hypothetical protein
TERG_05854	1.71	beta-lactamase (*T. tonsurans*)
TERG_05118	1.71	actin cytoskeleton organization protein (*T. tonsurans*)
TERG_04654	1.71	ubiquitin carboxyl-terminal hydrolase (*T. tonsurans*)
TERG_02243	1.70	hypothetical protein
TERG_07077	1.70	C6 transcription factor (*T. equinum*)
TERG_00313	1.70	hemolysin-III channel protein Izh2 (*T. tonsurans*)
TERG_03141	1.70	hypothetical protein
TERG_05554	1.70	integral membrane protein (*T. equinum*)
TERG_05851	1.69	hypothetical protein
TERG_01843	1.69	inositol-3-phosphate synthase (*T. tonsurans*)
TERG_05800	1.68	ubiquitin-protein ligase (Hul4), putative (*A. benhamiae*)
TERG_03332	1.68	mitochondrial carrier protein (*T. tonsurans*)
TERG_02016	1.68	Atg15p (*M. gypseum*)
TERG_07332	1.68	hypothetical protein

TERG_12324	1.68	hypothetical protein
TERG_00267	1.68	hypothetical protein
TERG_04703	1.68	DUF1715 domain-containing protein (*T. equinum*)
TERG_06247	1.67	DUF821 domain-containing protein (*T. equinum*)
TERG_08378	1.67	hypothetical protein
TERG_03709	1.67	hypothetical protein
TERG_00803	1.66	DUF907 domain protein (*A. benhamiae*)
TERG_03702	1.66	hypothetical protein
TERG_01153	1.66	hypothetical protein
TERG_04590	1.66	dihydrofolate synthetase Fol3 (*T. equinum*)
TERG_01618	1.66	hypothetical protein
TERG_06681	1.66	glycogen debranching enzyme
TERG_07307	1.66	hypothetical protein
TERG_08824	1.65	hypothetical protein
TERG_03182	1.65	serine/threonine protein kinase (*T. equinum*)
TERG_08381	1.65	NUDIX domain-containing protein (*T. equinum*)
TERG_06111	1.65	DUF1711 domain-containing protein (*M. canis*)
TERG_02816	1.64	dienelactone hydrolase (*T. equinum*)
TERG_08708	1.64	SesA protein (*T. equinum*)
TERG_06209	1.63	UTR2 protein (*M. gypseum*)
TERG_07224	1.63	hypothetical protein
TERG_06016	1.63	glycosyl hydrolase (*T. tonsurans*)
TERG_06675	1.63	GliK protein (*T. equinum*)
TERG_12629	1.63	NAD(P) transhydrogenase (*T. equinum*)
TERG_07567	1.63	DUF647 domain-containing protein (*T. equinum*)
TERG_04123	1.63	hypothetical protein
TERG_04103	1.63	hypothetical protein
TERG_03533	1.62	DRAP deaminase, putative (*A. benhamiae*)
TERG_02511	1.62	salicylate hydroxylase, putative (*A. benhamiae*)
TERG_07996	1.62	hypothetical protein
TERG_01292	1.62	glycosyl hydrolase, putative (*A. benhamiae*)
TERG_02843	1.62	C6 transcription factor (*T. equinum*)
TERG_01856	1.62	CMGC/SRPK protein kinase
TERG_08044	1.61	GPI anchored protein, putative (*A. benhamiae*)
TERG_05562	1.61	2-hydroxychromene-2-carboxylate isomerase, putative (*A. benhamiae*)
TERG_11885	1.61	ABC bile acid transporter, putative (*T. verrucosum*)
TERG_02481	1.61	F-box domain-containing protein (*M. canis*)
TERG_01996	1.60	serine/threonine protein kinase
TERG_06899	1.60	alkaline phosphatase (*T. tonsurans*)
TERG_00780	1.60	hypothetical protein
TERG_01105	1.60	serine/threonine protein kinase (*T. tonsurans*)
TERG_02567	1.60	phytanoyl-CoA dioxygenase (*T. tonsurans*)
TERG_02140	1.59	bZIP transcription factor (*T. tonsurans*)
TERG_11682	1.59	hypothetical protein
TERG_00860	1.59	LRP16 family protein (*T. tonsurans*)
TERG_02410	1.59	cytoskeleton assembly control protein Sla2 (*T. tonsurans*)
TERG_12057	1.58	hypothetical protein
TERG_08902	1.58	hypothetical protein
TERG_03408	1.58	ZIP metal ion transporter (*T. equinum*)

TERG_08811	1.58	hypothetical protein
TERG_11770	1.58	hypothetical protein
TERG_01841	1.58	hypothetical protein
TERG_07761	1.58	hypothetical protein
TERG_06765	1.58	ULK/ULK protein kinase
TERG_08287	1.58	ATP-citrate synthase (*T. equinum*)
TERG_02524	1.58	proline-specific permease (*T. equinum*)
TERG_03032	1.58	autophagy protein Atg13, putative (*A. benhamiae*)
TERG_02445	1.58	hypothetical protein
TERG_06294	1.57	V-SNARE (*T. equinum*)
TERG_04210	1.57	CAMKK/CAMKK-META protein kinase
TERG_07917	1.57	hypothetical protein
TERG_08066	1.57	STE/STE7 protein kinase
TERG_08011	1.57	metallocarboxypeptidase A-like protein
TERG_07707	1.57	C6 zinc finger domain-containing protein (*T. equinum*)
TERG_08878	1.57	hypothetical protein
TERG_01914	1.57	trichodiene oxygenase (*T. equinum*)
TERG_02550	1.57	ZZ type zinc finger domain protein (*T. verrucosum*)
TERG_05963	1.57	WD repeat protein (*T. tonsurans*)
TERG_06836	1.56	CAMK/CAMK1/CAMK1-RCK protein kinase
TERG_12636	1.56	hypothetical protein
TERG_04434	1.56	DNA repair protein
TERG_00349	1.55	hypothetical protein
TERG_06472	1.55	ketoreductase (*T. tonsurans*)
TERG_05813	1.55	1-phosphatidylinositol-3-phosphate 5-kinase (*T. equinum*)
TERG_11943	1.55	MFS maltose permease (*T. tonsurans*)
TERG_06533	1.55	glyoxalase family protein (*A. benhamiae*)
TERG_07706	1.54	hypothetical protein
TERG_01433	1.54	WD domain, G-beta repeat protein (*A. benhamiae*)
TERG_08427	1.54	C6 transcription factor, putative (*A. benhamiae*)
TERG_08288	1.54	ATP-citrate synthase subunit 1
TERG_12630	1.54	nicotinamide nucleotide transhydrogenase (*T. tonsurans*)
TERG_06884	1.54	C6 transcription factor (War1), putative (*T. verrucosum*)
TERG_01603	1.53	UBX domain-containing protein (*T. equinum*)
TERG_02087	1.53	hypothetical protein
TERG_05230	1.53	hypothetical protein
TERG_05552	1.53	DNA mismatch repair protein MutL (*T. tonsurans*)
TERG_07174	1.53	vesicle transport V-snare protein (*T. equinum*)
TERG_02527	1.53	WD40 repeat protein (*A. benhamiae*)
TERG_00097	1.53	hypothetical protein
TERG_03835	1.53	hypothetical protein
TERG_07223	1.53	hypothetical protein
TERG_00286	1.53	ABC transporter (*T. tonsurans*)
TERG_03200	1.53	DNA repair and recombination protein RAD26 (*T. tonsurans*)
TERG_05802	1.53	hypothetical protein
TERG_00580	1.52	7-dehydrocholesterol reductase (*T. equinum*)
TERG_00589	1.52	phosphoesterase (*T. tonsurans*)
TERG_00570	1.52	autophagy protein (*T. equinum*)
TERG_00969	1.52	hypothetical protein

TERG_05144	1.52	hypothetical protein
TERG_07086	1.52	hypothetical protein
TERG_01811	1.51	triacylglycerol lipase, putative (*A. benhamiae*)
TERG_00654	1.51	hypothetical protein
TERG_12393	1.51	hypothetical protein
TERG_11582	1.51	hypothetical protein
TERG_05580	1.51	hypothetical protein
TERG_08848	1.51	thioredoxin, putative (*A. benhamiae*)
TERG_05405	1.51	methionine permease (*T. equinum*)
TERG_02369	1.51	MFS transporter (*T. tonsurans*)
TERG_07492	1.50	sugar transporter STL1 (*T. tonsurans*)
TERG_06777	1.50	hypothetical protein
TERG_04414	1.50	isoflavone reductase family protein (CipA), putative (*A. benhamiae*)
TERG_07337	-1.50	54S ribosomal protein RML2 (*T. equinum*)
TERG_02122	-1.50	adenylate-forming enzyme, putative (*A. benhamiae*)
TERG_01643	-1.50	hypothetical protein
TERG_04382	-1.50	c-14 sterol reductase (*T. tonsurans*)
TERG_12344	-1.51	kinesin family protein (*T. tonsurans*)
TERG_07335	-1.51	G2/M-specific cyclin NimE (*T. tonsurans*)
TERG_08318	-1.51	HAD superfamily hydrolase (*T. tonsurans*)
TERG_05384	-1.51	hypothetical protein
TERG_00211	-1.51	proteasome subunit alpha type 6 (*T. equinum*)
TERG_07895	-1.51	prefoldin, alpha subunit
TERG_08991	-1.51	ATPase 2 nuclear control (*M. gypseum*)
TERG_02465	-1.51	aminoalcoholphosphotransferase (*T. equinum*)
TERG_04192	-1.51	lactonohydrolase, putative (*A. benhamiae*)
TERG_06122	-1.52	hypothetical protein
TERG_12392	-1.52	hypothetical protein
TERG_08675	-1.52	hypothetical protein
TERG_12355	-1.52	hypothetical protein
TERG_00524	-1.52	trans-sialidase (*M. canis*)
TERG_07157	-1.52	U3 small nucleolar RNA-associated protein 5 (*T. equinum*)
TERG_05366	-1.52	hypothetical protein
TERG_06976	-1.52	separin, putative (*T. verrucosum*)
TERG_04508	-1.52	AAA ATPase (*T. equinum*)
TERG_00170	-1.53	hypothetical protein
TERG_03642	-1.53	hypothetical protein
TERG_00008	-1.53	MFS phospholipid transporter (*T. tonsurans*)
TERG_00885	-1.53	60S ribosome biogenesis protein Rrp14 (*T. tonsurans*)
TERG_00004	-1.53	alanine aminotransferase (*T. tonsurans*)
TERG_02250	-1.53	cyclopropane-fatty-acyl-phospholipid synthase (*T. tonsurans*)
TERG_07587	-1.53	hypothetical protein
TERG_06305	-1.53	DNA-directed RNA polymerase I and III (*T. tonsurans*)
TERG_04087	-1.53	DnaJ domain-containing protein (*T. tonsurans*)
TERG_00192	-1.53	ATP-dependent rRNA helicase spb4 (*T. tonsurans*)
TERG_08845	-1.54	pyridoxamine phosphate oxidase (*T. tonsurans*)
TERG_03498	-1.54	pre-rRNA processing protein Tsr1 (*T. tonsurans*)
TERG_12121	-1.54	DNA replication licensing factor mcm5 (*T. tonsurans*)
TERG_12504	-1.54	MFS multidrug transporter (*T. equinum*)

TERG_00538	-1.54	oxidoreductase (*T. tonsurans*)
TERG_00819	-1.54	PE repeat family protein (*T. verrucosum*)
TERG_04586	-1.54	ribonucleoside-diphosphate reductase large chain
TERG_01761	-1.54	hypothetical protein
TERG_06366	-1.54	CAMK protein kinase
TERG_07799	-1.54	fatty acids protein 3 elongation (*T. equinum*)
TERG_05809	-1.55	hypothetical protein
TERG_12434	-1.55	hypothetical protein
TERG_12395	-1.55	hypothetical protein
TERG_04636	-1.55	4-hydroxybenzoate polyprenyl transferase
TERG_04378	-1.55	aldehyde reductase I (ARI), putative (*T. verrucosum*)
TERG_02037	-1.55	nucleic acid-binding protein (*A. benhamiae*)
TERG_06553	-1.55	hypothetical protein
TERG_07120	-1.56	hypothetical protein
TERG_02057	-1.56	hypothetical protein
TERG_06933	-1.56	hypothetical protein
TERG_11862	-1.56	integral membrane protein (*T. tonsurans*)
TERG_02176	-1.56	small nuclear ribonucleoprotein LSM2 (*T. equinum*)
TERG_05790	-1.56	HMG box protein, putative (*A. benhamiae*)
TERG_03174	-1.57	MFS siderochrome iron transporter MirB (*T. verrucosum*)
TERG_12340	-1.57	hypothetical protein
TERG_11847	-1.57	hypothetical protein
TERG_03256	-1.57	carbonate dehydratase (*T. equinum*)
TERG_01303	-1.57	hypothetical protein
TERG_04060	-1.57	C2H2 type zinc finger domain-containing protein (*T. equinum*)
TERG_01771	-1.57	FAD binding monooxygenase, putative (*T. verrucosum*)
TERG_04908	-1.57	signal recognition particle, putative (*A. benhamiae*)
TERG_03777	-1.57	hypothetical protein
TERG_06918	-1.58	hypothetical protein
TERG_07963	-1.58	hypothetical protein
TERG_00983	-1.58	eukaryotic translation initiation factor 3 subunit K
TERG_12053	-1.58	HEAT repeat protein (*T. verrucosum*)
TERG_07341	-1.58	DNA replication helicase Dna2 (*T. tonsurans*)
TERG_02076	-1.58	hypothetical protein
TERG_00372	-1.58	hypothetical protein
TERG_01231	-1.58	ribosomal large subunit biogenesis protein MAK16 (*T. tonsurans*)
TERG_02538	-1.59	carboxylesterase (*T. equinum*)
TERG_00386	-1.59	hypothetical protein
TERG_11809	-1.59	hydroxymethylglutaryl-CoA synthase
TERG_01812	-1.59	nucleolar complex protein 14 (*T. equinum*)
TERG_02788	-1.59	DNA-directed RNA polymerase I (*T. tonsurans*)
TERG_04349	-1.59	hypothetical protein
TERG_00894	-1.59	translation initiation factor IF-2
TERG_05370	-1.59	hypothetical protein
TERG_05254	-1.60	translation initiation factor RLI1
TERG_04684	-1.60	shugoshin family protein (*A. benhamiae*)
TERG_12200	-1.60	hypothetical protein
TERG_01697	-1.60	hypothetical protein
TERG_01239	-1.60	Mis6 domain-containing protein (*T. equinum*)

TERG_08234	-1.60	TCTP family protein (*T. verrucosum*)
TERG_07613	-1.60	nuclear envelope protein (*T. equinum*)
TERG_03345	-1.60	hypothetical protein
TERG_01443	-1.61	ABC multidrug transporter (*T. tonsurans*)
TERG_07061	-1.61	CMGC/SRPK protein kinase
TERG_02293	-1.61	hypothetical protein
TERG_02393	-1.61	hypothetical protein
TERG_02575	-1.61	hypothetical protein
TERG_02973	-1.61	morphogenesis protein (Msb1), putative (*T. verrucosum*)
TERG_02854	-1.61	dynein light chain (*T. tonsurans*)
TERG_11721	-1.61	acyl-CoA dehydrogenase (*T. tonsurans*)
TERG_06144	-1.61	cell wall serine-threonine-rich galactomannoprotein Mp1 (*A. benhamiae*)
TERG_06129	-1.61	60S ribosomal protein
TERG_11758	-1.62	DUF866 domain-containing protein (*T. equinum*)
TERG_03084	-1.62	pentatricopeptide repeat protein (*A. benhamiae*)
TERG_01507	-1.62	extracellular proline-glycine rich protein (*T. verrucosum*)
TERG_05388	-1.62	ribosome biogenesis protein (*T. tonsurans*)
TERG_04410	-1.62	hypothetical protein
TERG_06230	-1.62	hypothetical protein
TERG_04819	-1.62	Fe superoxide dismutase (*T. tonsurans*)
TERG_05701	-1.62	C4-dicarboxylate transporter/malic acid transport protein, putative (*T. verrucosum*)
TERG_00249	-1.62	ATP-dependent rRNA helicase RRP3
TERG_06621	-1.62	60S ribosomal subunit assembly/export protein loc1 (*T. tonsurans*)
TERG_05967	-1.63	hypothetical protein
TERG_02590	-1.63	peptidyl-prolyl cis-trans isomerase D
TERG_02251	-1.63	hypothetical protein
TERG_00974	-1.63	2,3-bisphosphoglycerate-independent phosphoglycerate mutase
TERG_04062	-1.63	low-temperature viability protein ltv1 (*T. tonsurans*)
TERG_06964	-1.63	NTF2 domain-containing protein (*T. tonsurans*)
TERG_02326	-1.63	WD domain-containing protein (*T. tonsurans*)
TERG_02616	-1.63	integral membrane protein (*T. equinum*)
TERG_05846	-1.63	G1/S-specific cyclin CLN1 (*T. equinum*)
TERG_01257	-1.63	nascent polypeptide-associated complex subunit alpha
TERG_08884	-1.63	GPI-anchored cell surface glycoprotein, putative (*T. verrucosum*)
TERG_06528	-1.64	Delta(24(24(1)))-sterol reductase (*T. equinum*)
TERG_08429	-1.64	eukaryotic translation initiation factor 3 subunit H
TERG_00893	-1.64	mitochondrial carrier protein, putative (*T. verrucosum*)
TERG_05434	-1.64	ribosome associated DnaJ chaperone Zuotin (*T. equinum*)
TERG_06375	-1.64	hypothetical protein
TERG_01959	-1.64	RNA polymerase Rpb1 C-terminal repeat domain-containing protein (*T. equinum*)
TERG_01679	-1.64	Trm112p-like protein (*T. equinum*)
TERG_12131	-1.64	alcohol dehydrogenase (*T. tonsurans*)
TERG_05891	-1.65	MFS phosphate transporter (*T. equinum*)
TERG_12325	-1.65	hypothetical protein
TERG_08623	-1.65	O-methyltransferase GliM2 (*T. equinum*)
TERG_08443	-1.66	adenosylhomocysteinase
TERG_04307	-1.66	hypothetical protein
TERG_03339	-1.66	alternative oxidase, mitochondrial

TERG_03587	-1.66	WD repeat protein (*T. verrucosum*)
TERG_07444	-1.66	nuclear segregation protein (*T. tonsurans*)
TERG_05002	-1.67	UDP-glucose 4-epimerase GalE
TERG_05925	-1.67	EF-hand protein (*T. tonsurans*)
TERG_03205	-1.67	DNA polymerase alpha catalytic subunit (*T. equinum*)
TERG_12620	-1.67	hypothetical protein
TERG_00757	-1.68	hypothetical protein
TERG_07557	-1.68	hypothetical protein
TERG_04339	-1.68	signal transduction protein Syg1, putative (*A. benhamiae*)
TERG_07473	-1.68	DNA-directed RNA polymerase I (*T. tonsurans*)
TERG_06744	-1.68	fructose-bisphosphate aldolase 1
TERG_00124	-1.68	peroxisomal matrix protein (*T. equinum*)
TERG_07131	-1.68	phosphotransmitter protein Ypd1 (*T. equinum*)
TERG_03453	-1.68	eukaryotic translation initiation factor 5 (*T. tonsurans*)
TERG_01968	-1.68	RNA binding protein, putative (*T. verrucosum*)
TERG_05948	-1.68	protein kinase subdomain-containing protein (*T. equinum*)
TERG_06358	-1.68	dicer (*T. tonsurans*)
TERG_04131	-1.68	heat shock trehalose synthase, putative (*T. verrucosum*)
TERG_03270	-1.68	small nucleolar ribonucleoprotein complex subunit (*T. tonsurans*)
TERG_07616	-1.68	diphosphomevalonate decarboxylase
TERG_04995	-1.68	ribosome biogenesis protein Urb1, putative (*A. benhamiae*)
TERG_01310	-1.69	nascent polypeptide-associated complex subunit beta
TERG_07024	-1.69	endoribonuclease L-PSP (*T. tonsurans*)
TERG_08368	-1.69	TTK protein kinase
TERG_08497	-1.69	mRNA turnover protein 4 (*T. equinum*)
TERG_05558	-1.69	ornithine decarboxylase, putative (*T. verrucosum*)
TERG_11915	-1.69	nucleolus protein required for cell viability (*T. tonsurans*)
TERG_05956	-1.69	rRNA processing protein Ebp2p (*T. equinum*)
TERG_06150	-1.69	deoxyuridine 5'-triphosphate nucleotidohydrolase
TERG_01206	-1.69	pre-mRNA-splicing factor ATP-dependent RNA helicase PRP43 (*T. equinum*)
TERG_06662	-1.70	eukaryotic translation initiation factor 3 subunit B
TERG_06578	-1.70	glutathione S-transferase, putative (*A. benhamiae*)
TERG_02617	-1.70	DNA primase subunit Pri1 (*T. tonsurans*)
TERG_04959	-1.70	hypothetical protein
TERG_03124	-1.70	histone
TERG_00977	-1.70	hypothetical protein
TERG_00703	-1.70	nuclear migration protein, putative (*A. benhamiae*)
TERG_03172	-1.71	sodium/phosphate symporter (*T. tonsurans*)
TERG_03499	-1.71	U3 small nucleolar RNA-associated protein 7 (*T. equinum*)
TERG_00834	-1.71	60S ribosomal protein L24b (*T. equinum*)
TERG_12503	-1.71	polyamine transporter 2
TERG_01612	-1.71	enolase
TERG_00373	-1.71	hypothetical protein
TERG_08755	-1.71	M-phase inducer phosphatase (*T. tonsurans*)
TERG_05261	-1.72	karyopherin alpha subunit (*T. tonsurans*)
TERG_04402	-1.72	glyceraldehyde-3-phosphate dehydrogenase
TERG_07361	-1.72	hybrid PKS-NRPS enzyme, putative (*A. benhamiae*)
TERG_01957	-1.72	proteinase T (*M. gypseum*)
TERG_04746	-1.72	nucleolar GTP-binding protein (*T. tonsurans*)

TERG_07539	-1.72	multidrug resistance protein (*T. tonsurans*)
TERG_02319	-1.72	metallo-beta-lactamase superfamily protein (*T. equinum*)
TERG_05242	-1.73	PUF6 (*M. gypseum*)
TERG_02772	-1.73	DNA polymerase V (*T. tonsurans*)
TERG_07958	-1.73	myosin class II heavy chain (MHC), putative (*T. verrucosum*)
TERG_02023	-1.73	extracellular matrix protein, putative (*T. verrucosum*)
TERG_01787	-1.73	integral membrane protein (*T. equinum*)
TERG_08918	-1.73	hypothetical protein
TERG_12373	-1.73	multidrug resistance protein (*T. tonsurans*)
TERG_07547	-1.73	RGS domain-containing protein (*T. tonsurans*)
TERG_03625	-1.74	hypothetical protein
TERG_07736	-1.74	hypothetical protein
TERG_00573	-1.75	hypothetical protein
TERG_03497	-1.75	hemolysin-III family protein (*T. equinum*)
TERG_12631	-1.75	conserved lysine-rich protein, putative (*T. verrucosum*)
TERG_05865	-1.76	integral membrane protein, Mpv17/PMP22 family, putative (*T. verrucosum*)
TERG_08125	-1.76	nucleolar complex protein 2 (*T. equinum*)
TERG_02866	-1.76	ribosome biogenesis protein ERB1
TERG_01377	-1.76	hypothetical protein
TERG_07641	-1.76	GPI-anchored cell wall protein Pst1, putative (*A. benhamiae*)
TERG_04616	-1.76	hypothetical protein
TERG_06906	-1.76	DNA mismatch repair protein msh6 (*T. equinum*)
TERG_01046	-1.76	H/ACA ribonucleoprotein complex subunit 1
TERG_00257	-1.77	conserved predicted protein (*A. benhamiae*)
TERG_01010	-1.77	Leucine Rich Repeat domain protein (*A. benhamiae*)
TERG_00181	-1.77	nucleolar protein 58
TERG_11525	-1.77	hypothetical protein
TERG_05074	-1.77	NADP-dependent L-serine/L-allo-threonine dehydrogenase ydfG (*M. gypseum*)
TERG_05941	-1.78	stress responsive A/B barrel domain-containing protein (*T. equinum*)
TERG_11600	-1.78	MFS transporter (*T. tonsurans*)
TERG_12447	-1.78	F-box domain-containing protein (*M. gypseum*)
TERG_08391	-1.78	hypothetical protein
TERG_02399	-1.78	RNA-directed RNA polymerase (*T. tonsurans*)
TERG_00456	-1.78	cysteine dioxygenase, putative (*A. benhamiae*)
TERG_11907	-1.78	FAD binding domain containing protein (*T. equinum*)
TERG_08289	-1.78	Rpp14 family protein (*T. equinum*)
TERG_08236	-1.79	hypothetical protein
TERG_04964	-1.79	RNA-binding protein (*A. benhamiae*)
TERG_02888	-1.80	PWI domain mRNA processing protein, putative (*T. verrucosum*)
TERG_00117	-1.80	SNF2 family helicase, putative (*T. verrucosum*)
TERG_03935	-1.80	hypothetical protein
TERG_06810	-1.80	hypothetical protein
TERG_12632	-1.80	conserved lysine-rich protein, putative (*A. benhamiae*)
TERG_06158	-1.81	60S ribosomal protein L29
TERG_04790	-1.81	arsenical-resistance protein
TERG_03977	-1.81	hypothetical protein
TERG_01440	-1.81	vegetative cell wall protein gp1 (*M. canis*)
TERG_11869	-1.81	cysteine protease atg4 (*T. equinum*)
TERG_00063	-1.82	lysine-tRNA ligase

TERG_12119	-1.82	hypothetical protein
TERG_06264	-1.82	steroid monooxygenase, putative (*T. verrucosum*)
TERG_06222	-1.82	MYB DNA-binding domain-containing protein (*T. equinum*)
TERG_01591	-1.82	small nucleolar ribonucleoprotein complex subunit (*T. equinum*)
TERG_06844	-1.82	40S ribosomal protein S21
TERG_02058	-1.82	ribosome biogenesis protein RPF2 (*T. equinum*)
TERG_03364	-1.83	hypothetical protein
TERG_03102	-1.83	sterol 24-C-methyltransferase (*T. equinum*)
TERG_12633	-1.83	conserved lysine-rich protein, putative (*A. benhamiae*)
TERG_12526	-1.83	hypothetical protein
TERG_00605	-1.83	imidazoleglycerol-phosphate dehydratase (*T. equinum*)
TERG_12059	-1.83	transporter smf2 (*T. tonsurans*)
TERG_00640	-1.83	eukaryotic translation initiation factor 3 subunit L
TERG_01853	-1.84	hypothetical protein
TERG_07047	-1.84	cholestenol delta-isomerase, putative (*T. verrucosum*)
TERG_07939	-1.84	ribosomal RNA processing protein (*T. equinum*)
TERG_06090	-1.84	C2H2 transcription factor (TFIIIA), putative (*T. verrucosum*)
TERG_03734	-1.84	pre-rRNA processing protein Rrp12 (*T. tonsurans*)
TERG_01490	-1.84	LysR family regulatory protein (*M. gypseum*)
TERG_12252	-1.85	GTP-binding protein (*T. tonsurans*)
TERG_02914	-1.85	tRNA (guanine-N(7)-)-methyltransferase
TERG_12390	-1.85	hypothetical protein
TERG_01076	-1.85	aconitate hydratase, mitochondrial
TERG_00922	-1.85	meiotically up-regulated 65 protein (*T. equinum*)
TERG_04893	-1.85	polyadenylate-binding protein, cytoplasmic and nuclear
TERG_11948	-1.85	hypothetical protein
TERG_08655	-1.86	ADP,ATP carrier protein
TERG_02139	-1.86	eukaryotic translation initiation factor 3 subunit D
TERG_08961	-1.86	hypothetical protein
TERG_11690	-1.86	protein kinase subdomain-containing protein (*T. tonsurans*)
TERG_04002	-1.86	ribosome assembly protein SQT1 (*T. equinum*)
TERG_07810	-1.86	phospholipase (*T. tonsurans*)
TERG_04437	-1.87	nucleolar protein NOP2 (*T. tonsurans*)
TERG_07171	-1.87	hypothetical protein
TERG_07125	-1.87	peptidyl-tRNA hydrolase
TERG_06651	-1.87	cytochrome P450, putative (*T. verrucosum*)
TERG_00720	-1.87	60S ribosomal protein L19 (*T. tonsurans*)
TERG_07409	-1.87	amino acid permease (*T. equinum*)
TERG_00105	-1.87	40S ribosomal protein S30
TERG_04875	-1.88	monocarboxylate permease (*T. equinum*)
TERG_06917	-1.88	elongation factor G, mitochondrial
TERG_01372	-1.88	hypothetical protein
TERG_05021	-1.88	C6 transcription factor, putative (*A. benhamiae*)
TERG_05146	-1.88	protein methyltransferase (*T. tonsurans*)
TERG_04025	-1.88	hypothetical protein
TERG_07882	-1.89	myosin type II heavy chain (*T. tonsurans*)
TERG_06522	-1.89	FMN dependent dehydrogenase, putative (*T. verrucosum*)
TERG_06893	-1.89	aspartyl-tRNA synthetase (*T. tonsurans*)
TERG_02125	-1.89	hypothetical protein

TERG_07947	-1.89	Sas10/Utp3 family protein (*T. tonsurans*)
TERG_03225	-1.89	betaine aldehyde dehydrogenase, putative (*A. benhamiae*)
TERG_04899	-1.89	ubiquitin-60S ribosomal protein L40
TERG_06539	-1.90	fructosamine-3-kinase (*T. equinum*)
TERG_06026	-1.90	ribosomal protein S24 (*T. tonsurans*)
TERG_04979	-1.90	RNA exonuclease 4 (*T. tonsurans*)
TERG_01494	-1.90	hypothetical protein
TERG_02921	-1.90	hypothetical protein
TERG_07362	-1.90	UV damage endonuclease UvdE
TERG_04676	-1.91	cell division control protein 10 (*T. tonsurans*)
TERG_05039	-1.91	ribonucleoprotein-associated protein
TERG_05342	-1.91	MFS multidrug transporter, putative (*A. benhamiae*)
TERG_02867	-1.91	ribosome biogenesis ATPase RIX7 (*T. tonsurans*)
TERG_07950	-1.91	translation elongation factor 1 subunit Eef1-beta (*T. equinum*)
TERG_05799	-1.91	chlorophyll synthesis pathway protein BchC
TERG_06796	-1.91	kinesin family protein (*T. equinum*)
TERG_00962	-1.91	protein kinase subdomain-containing protein (*T. tonsurans*)
TERG_12199	-1.91	hypothetical protein
TERG_06032	-1.91	ribosomal small subunit assembly protein, putative (*A. benhamiae*)
TERG_07922	-1.92	glycine cleavage system T protein
TERG_03400	-1.92	subtilisin-like protease 1
TERG_12501	-1.92	catechol dioxygenase, putative (*T. verrucosum*)
TERG_12294	-1.92	histidine acid phosphatase, putative (*T. verrucosum*)
TERG_12533	-1.93	hypothetical protein
TERG_06218	-1.93	60S ribosomal protein L34 (*T. equinum*)
TERG_05278	-1.93	microtubule associated protein (*T. tonsurans*)
TERG_12120	-1.93	DNA replication licensing factor mcm5
TERG_12491	-1.93	carboxylesterase, putative (*A. benhamiae*)
TERG_08363	-1.93	tyrosinase (*T. verrucosum*)
TERG_04238	-1.93	H/K ATPase alpha subunit (*T. equinum*)
TERG_02587	-1.94	hypothetical protein
TERG_07059	-1.94	hypothetical protein
TERG_08794	-1.94	pyruvate dehydrogenase kinase (*T. equinum*)
TERG_00708	-1.94	serine hydroxymethyltransferase (*T. tonsurans*)
TERG_04548	-1.94	SH3 domain-containing protein cyk3 (*T. equinum*)
TERG_06109	-1.94	RNA polymerase I subunit Rpa43, putative (*T. verrucosum*)
TERG_12511	-1.95	MFS multidrug transporter (*T. equinum*)
TERG_02672	-1.95	ATP-dependent RNA helicase has1
TERG_11979	-1.95	cell division control protein 54 (*T. tonsurans*)
TERG_03401	-1.95	high affinity nickel transporter (*T. tonsurans*)
TERG_12321	-1.96	hypothetical protein
TERG_06172	-1.96	60S ribosomal protein L12
TERG_00943	-1.96	DNA mismatch repair protein msh-2 (*M. canis*)
TERG_08269	-1.97	polysaccharide deacetylase (*T. tonsurans*)
TERG_07012	-1.97	rRNA 2'-O-methyltransferase fibrillarin
TERG_12602	-1.97	4-coumarate-CoA ligase (*T. equinum*)
TERG_08133	-1.97	40S ribosomal protein S25
TERG_08990	-1.97	hypothetical protein
TERG_08375	-1.97	ATP-dependent RNA helicase eIF4A

TERG_01446	-1.97	GMP synthase [glutamine-hydrolyzing]
TERG_12226	-1.97	SNF2 family helicase (*T. equinum*)
TERG_05685	-1.97	60S ribosomal protein L13 (*T. tonsurans*)
TERG_00679	-1.97	AhpC/TSA family protein (*T. equinum*)
TERG_00550	-1.98	hypothetical protein
TERG_00633	-1.98	40S ribosomal protein S16
TERG_07477	-1.98	copper-sulfate regulated protein 1 (*T. equinum*)
TERG_04450	-1.98	60S ribosomal protein L23 (*T. tonsurans*)
TERG_02640	-1.98	pre-rRNA processing protein Utp22 (*T. verrucosum*)
TERG_02759	-1.98	hexokinase XprF (*T. equinum*)
TERG_11645	-1.99	Phytanoyl-CoA dioxygenase PhyH (*T. equinum*)
TERG_12682	-1.99	hypothetical protein
TERG_11558	-1.99	hypothetical protein
TERG_08334	-2.00	proline oxidase Put1, putative (*A. benhamiae*)
TERG_05363	-2.00	hypothetical protein
TERG_11685	-2.00	hypothetical protein
TERG_02397	-2.00	60S ribosomal protein L37
TERG_06365	-2.00	ribosomal protein L15
TERG_05511	-2.00	AT DNA binding protein (*T. equinum*)
TERG_00531	-2.00	40S ribosomal protein S7
TERG_12259	-2.00	CMGC/CLK protein kinase (*T. equinum*)
TERG_04281	-2.01	woronin body major protein
TERG_04043	-2.01	hypothetical protein
TERG_08924	-2.01	MIZ zinc finger domain protein (*A. benhamiae*)
TERG_02322	-2.02	hypothetical protein
TERG_05401	-2.02	rRNA processing protein Bystin (*T. tonsurans*)
TERG_04924	-2.02	threonine aldolase, putative (*T. verrucosum*)
TERG_03672	-2.02	hypothetical protein
TERG_04647	-2.02	60S ribosomal protein L18 (*T. equinum*)
TERG_07626	-2.02	hypothetical protein
TERG_08545	-2.02	C-4 methyl sterol oxidase Erg25 (*T. equinum*)
TERG_01125	-2.03	SprT family metallopeptidase, putative (*T. verrucosum*)
TERG_05003	-2.03	NmrA family protein (*T. equinum*)
TERG_08672	-2.03	HFR-3 (*M. canis*)
TERG_08722	-2.04	hypothetical protein
TERG_04411	-2.04	dipeptidase (*T. equinum*)
TERG_12263	-2.05	hypothetical protein
TERG_06262	-2.05	mitochondrial carrier protein (*T. tonsurans*)
TERG_00881	-2.05	zinc-containing alcohol dehydrogenase, putative (*T. verrucosum*)
TERG_11906	-2.06	FAD binding domain containing protein (*T. equinum*)
TERG_06760	-2.06	ATP binding L-PSP endoribonuclease family protein, putative (*T. verrucosum*)
TERG_07231	-2.06	hemolysin-III family protein (*T. equinum*)
TERG_02237	-2.06	40S ribosomal protein S28
TERG_04130	-2.06	plasma membrane ATPase
TERG_00106	-2.07	60S ribosomal protein L24
TERG_07999	-2.07	hypothetical protein
TERG_12334	-2.07	hypothetical protein
TERG_01952	-2.07	40S ribosomal protein S15
TERG_11646	-2.07	hypothetical protein

TERG_07385	-2.07	60S acidic ribosomal protein P1
TERG_05046	-2.07	hypothetical protein
TERG_01149	-2.07	choline dehydrogenase, putative (*T. verrucosum*)
TERG_00738	-2.08	60S ribosomal protein L44
TERG_06275	-2.08	40S ribosomal protein
TERG_04478	-2.08	40S ribosomal protein S1
TERG_00088	-2.08	beta-xylosidase, putative (*A. benhamiae*)
TERG_03929	-2.08	hypothetical protein
TERG_01415	-2.08	ribosomal protein L24
TERG_06846	-2.08	inosine-5'-monophosphate dehydrogenase
TERG_01779	-2.08	sentrin-specific protease (*T. equinum*)
TERG_00880	-2.08	actin filament organization protein App1-like, putative (*T. verrucosum*)
TERG_03624	-2.09	SUN domain protein (Uth1), putative (*T. verrucosum*)
TERG_06261	-2.09	phosphoesterase (*T. equinum*)
TERG_08282	-2.09	hypothetical protein
TERG_07509	-2.09	serine/threonine protein kinase (*M. canis*)
TERG_02260	-2.10	hypothetical protein
TERG_06236	-2.10	40S ribosomal protein S19
TERG_02542	-2.10	integral membrane protein Pth11-like, putative (*T. verrucosum*)
TERG_02861	-2.10	40S ribosomal protein S23
TERG_11916	-2.11	nucleolus protein required for cell viability (*T. tonsurans*)
TERG_01256	-2.11	ribosomal protein L14 (*T. tonsurans*)
TERG_12251	-2.11	GTP-binding protein (*T. tonsurans*)
TERG_06815	-2.11	bZIP transcription factor (*T. tonsurans*)
TERG_04073	-2.11	glutathione synthetase
TERG_02024	-2.12	hypothetical protein
TERG_04778	-2.12	ribosome assembly and transport protein Srp40, putative (*T. verrucosum*)
TERG_00491	-2.12	hypothetical protein
TERG_02631	-2.12	histone H2B
TERG_05737	-2.13	hypothetical protein
TERG_02231	-2.13	ribosome biogenesis protein (*T. tonsurans*)
TERG_07380	-2.13	40S ribosomal protein S26E
TERG_03116	-2.13	zf-PARP-type zinc finger protein (*T. tonsurans*)
TERG_00221	-2.13	hypothetical protein
TERG_06874	-2.14	UTP-glucose-1-phosphate uridylyltransferase (*T. tonsurans*)
TERG_07726	-2.14	hypothetical protein
TERG_02545	-2.14	MFS monocarboxylate transporter (*T. equinum*)
TERG_08821	-2.14	ribosome biogenesis (*T. equinum*)
TERG_02385	-2.14	hypothetical protein
TERG_08047	-2.14	hypothetical protein
TERG_05236	-2.14	60S ribosomal protein L35
TERG_06265	-2.14	LPS glycosyltransferase (*T. equinum*)
TERG_02611	-2.14	WD repeat protein (*A. benhamiae*)
TERG_04740	-2.15	ergosterol biosynthesis protein Erg28 (*T. tonsurans*)
TERG_06512	-2.15	hypothetical protein
TERG_06640	-2.15	phosphoribosylamine-glycine ligase
TERG_03880	-2.15	60S ribosomal protein L5
TERG_02886	-2.15	hypothetical protein
TERG_01018	-2.15	monooxygenase, putative (*A. benhamiae*)

TERG_05801	-2.15	60S ribosomal protein L15
TERG_00650	-2.15	40S ribosomal protein S17-A
TERG_07135	-2.15	hypothetical protein
TERG_02263	-2.16	hypothetical protein
TERG_07383	-2.16	aconitate hydratase, mitochondrial
TERG_04506	-2.16	60S ribosomal protein L4-A (*T. tonsurans*)
TERG_07066	-2.17	40S ribosomal protein S5
TERG_05424	-2.17	hypothetical protein
TERG_02283	-2.18	MFS transporter, putative (*T. verrucosum*)
TERG_05723	-2.18	hypothetical protein
TERG_06221	-2.18	CTP synthase
TERG_07072	-2.18	ATP-dependent RNA helicase DRS1
TERG_08771	-2.18	extracellular serine-threonine rich protein (*T. tonsurans*)
TERG_05626	-2.18	chitinase (*T. equinum*)
TERG_02534	-2.18	hypothetical protein
TERG_11688	-2.18	nitrilase (*T. tonsurans*)
TERG_06421	-2.18	hypothetical protein
TERG_07154	-2.18	iron donor protein CyaY
TERG_01718	-2.19	cell division control protein Cdc6 (*T. tonsurans*)
TERG_01604	-2.19	60S ribosomal protein L36
TERG_08095	-2.19	MFS multidrug transporter, putative (*A. benhamiae*)
TERG_04040	-2.19	Spo12 family protein (*T. tonsurans*)
TERG_11551	-2.19	telomere and ribosome associated protein Stm1 (*T. tonsurans*)
TERG_06437	-2.20	ribosomal protein L28e (*T. equinum*)
TERG_05345	-2.20	60S ribosomal protein L43
TERG_02404	-2.20	40S ribosomal protein S4
TERG_02496	-2.20	histone H1 (*T. tonsurans*)
TERG_05625	-2.20	class V chitinase, putative (*T. verrucosum*)
TERG_08533	-2.20	poly(A) polymerase Cid1 (*T. equinum*)
TERG_01419	-2.20	protein pyrABCN
TERG_04310	-2.20	alcohol dehydrogenase (*T. tonsurans*)
TERG_02895	-2.21	60S ribosomal protein L9-B (*T. equinum*)
TERG_05283	-2.21	60S ribosomal protein L32
TERG_07493	-2.21	Zinc knuckle transcription factor (CnjB) (*T. tonsurans*)
TERG_02953	-2.21	hypothetical protein
TERG_07235	-2.21	fructose-1,6-bisphosphatase (*T. tonsurans*)
TERG_07630	-2.21	FMN dependent dehydrogenase, putative (*T. verrucosum*)
TERG_00242	-2.22	60S ribosomal protein L27-A (*T. tonsurans*)
TERG_02118	-2.22	GPI anchored protein, putative (*A. benhamiae*)
TERG_03747	-2.22	phospholipase A2 (*T. equinum*)
TERG_12607	-2.22	ThiF domain-containing protein (*T. tonsurans*)
TERG_12500	-2.22	sorbitol dehydrogenase (*T. equinum*)
TERG_04576	-2.22	CRAL/TRIO domain protein (*T. verrucosum*)
TERG_12619	-2.22	hypothetical protein
TERG_04772	-2.23	40S ribosomal protein S8-B
TERG_01995	-2.23	hypothetical protein
TERG_08428	-2.23	hypothetical protein
TERG_07182	-2.23	40S ribosomal protein S6-B
TERG_00383	-2.24	60S ribosomal protein L16

TERG_03738	-2.24	60S ribosomal protein L3
TERG_07363	-2.24	hypothetical protein
TERG_02376	-2.25	60S ribosomal protein (*T. equinum*)
TERG_00103	-2.25	hypothetical protein
TERG_07988	-2.25	60S ribosomal protein L10a
TERG_06626	-2.25	hypothetical protein
TERG_02630	-2.25	histone H2A
TERG_12027	-2.25	hypothetical protein
TERG_05184	-2.25	glycine cleavage system H protein
TERG_06414	-2.26	DUF1338 domain-containing protein (*T. equinum*)
TERG_06475	-2.26	zinc knuckle domain-containing protein (*T. tonsurans*)
TERG_06755	-2.26	C-8 sterol isomerase (Erg-1), putative (*T. verrucosum*)
TERG_01838	-2.26	LIM domain protein (*A. benhamiae*)
TERG_07944	-2.26	60S ribosomal protein L6 (*T. equinum*)
TERG_12314	-2.26	hypothetical protein
TERG_03619	-2.27	transcription factor Tos4 (*T. equinum*)
TERG_05655	-2.27	AN1 zinc finger protein (*T. equinum*)
TERG_06952	-2.27	rRNA processing protein Pwp1 (*T. tonsurans*)
TERG_04775	-2.27	beta-N-hexosaminidase, putative (*A. benhamiae*)
TERG_07127	-2.27	homocitrate synthase, mitochondrial
TERG_11753	-2.28	MFS transporter (*T. tonsurans*)
TERG_07957	-2.28	hypothetical protein
TERG_05251	-2.28	phosphoglycerate kinase
TERG_01638	-2.28	hypothetical protein
TERG_05866	-2.28	40S ribosomal protein S2
TERG_02971	-2.28	60S ribosomal protein L35a (*T. equinum*)
TERG_01912	-2.29	MFS transporter (*T. equinum*)
TERG_01953	-2.29	60S acidic ribosomal protein P2
TERG_06824	-2.30	60S ribosomal protein L23
TERG_01336	-2.30	MFS transporter (*T. equinum*)
TERG_02937	-2.30	importin beta-4 subunit (*T. tonsurans*)
TERG_07904	-2.31	tubulin beta chain
TERG_07357	-2.31	hypothetical protein
TERG_05825	-2.31	40S ribosomal protein S11
TERG_06101	-2.31	ribosomal protein P0 (*T. equinum*)
TERG_04089	-2.32	RfxA protein (*T. equinum*)
TERG_03494	-2.32	hypothetical protein
TERG_08667	-2.32	hypothetical protein
TERG_08843	-2.32	hypothetical protein
TERG_04606	-2.32	general amino acid permease AGP2 (*T. equinum*)
TERG_04441	-2.33	centromere/microtubule-binding protein CBF5
TERG_08390	-2.33	hypothetical protein
TERG_01102	-2.33	60S ribosomal protein L8-B
TERG_02197	-2.33	nitrilase, putative (*T. verrucosum*)
TERG_04036	-2.34	adenosine kinase (*T. tonsurans*)
TERG_03340	-2.35	oxidoreductase (*T. equinum*)
TERG_07002	-2.35	60S ribosomal protein L17
TERG_04153	-2.35	60S ribosome biogenesis protein Brx1 (*T. tonsurans*)
TERG_02878	-2.35	eukaryotic translation initiation factor 3 subunit F

TERG_02377	-2.36	40S ribosomal protein S9
TERG_03204	-2.36	60S ribosomal protein L7
TERG_05544	-2.36	plk/plk-unclassified protein kinase (*T. tonsurans*)
TERG_08090	-2.36	plasma membrane iron permease (*T. tonsurans*)
TERG_01368	-2.36	Ribosomal protein L7Ae/L30e/S12e/Gadd45 family protein (*T. equinum*)
TERG_08254	-2.37	40S ribosomal protein S18
TERG_05893	-2.37	CMGC protein kinase (*T. tonsurans*)
TERG_07876	-2.37	hypothetical protein
TERG_00635	-2.38	40S ribosomal protein S14
TERG_00956	-2.38	extracellular thaumatin domain-containing protein (*T. equinum*)
TERG_12058	-2.38	transporter smf2 (*T. tonsurans*)
TERG_12358	-2.38	DNA repair protein Rad5 (*T. tonsurans*)
TERG_05756	-2.39	calmodulin-binding protein Sha1, putative (*T. verrucosum*)
TERG_07504	-2.39	sialidase (*T. tonsurans*)
TERG_06163	-2.39	MYB family conidiophore development protein FlbD (*T. equinum*)
TERG_08333	-2.39	1-pyrroline-5-carboxylate dehydrogenase
TERG_06246	-2.39	extracellular serine-threonine rich protein (*A. benhamiae*)
TERG_01634	-2.40	MFS multidrug transporter, putative (*T. verrucosum*)
TERG_00783	-2.40	guanine nucleotide-binding protein subunit beta-like protein
TERG_00462	-2.41	40S ribosomal protein
TERG_06496	-2.41	coproporphyrinogen III oxidase (*T. tonsurans*)
TERG_01066	-2.42	DNA methyltransferase Dim-2 (*T. equinum*)
TERG_11751	-2.42	PCI domain-containing protein (*T. tonsurans*)
TERG_02604	-2.42	40S ribosomal protein S20
TERG_03343	-2.43	fatty acid desaturase (*T. equinum*)
TERG_06266	-2.43	hypothetical protein
TERG_11740	-2.44	hypothetical protein
TERG_06315	-2.44	integral membrane protein (*A. benhamiae*)
TERG_12195	-2.44	developmental regulator flbA (*M. gypseum*)
TERG_02530	-2.46	hypothetical protein
TERG_11945	-2.46	hypothetical protein
TERG_00217	-2.47	ubiquitin-40S ribosomal protein S27a
TERG_02521	-2.47	60S ribosomal protein L27a
TERG_07029	-2.47	hypothetical protein
TERG_11760	-2.48	HIT finger domain protein, putative (*T. verrucosum*)
TERG_01985	-2.48	hypothetical protein
TERG_08108	-2.48	40S ribosomal protein S22
TERG_01550	-2.49	60S ribosomal protein L38 (*T. tonsurans*)
TERG_02189	-2.49	tubulin alpha-2 chain
TERG_12231	-2.49	DUF221 domain-containing protein (*T. equinum*)
TERG_05998	-2.49	chlorophyll synthesis pathway protein BchC
TERG_12033	-2.49	atypical/RIO/RIO1 protein kinase (*T. tonsurans*)
TERG_03780	-2.49	pyruvate dehydrogenase dihydrolipoamide acetyltransferase component (*M. gypseum*)
TERG_00504	-2.49	hypothetical protein
TERG_05788	-2.50	60S ribosomal protein L18A (*T. tonsurans*)
TERG_12318	-2.50	chitin synthase (*T. tonsurans*)
TERG_08273	-2.52	zinc finger protein ZPR1 (*T. tonsurans*)
TERG_06585	-2.52	hypothetical protein

TERG_03758	-2.52	hypothetical protein
TERG_07689	-2.53	ribosomal biogenesis protein Gar2 (*T. tonsurans*)
TERG_07069	-2.53	carbonic anhydrase (*T. tonsurans*)
TERG_00976	-2.54	serine hydroxymethyltransferase, cytosolic
TERG_05627	-2.54	LysM domain-containing protein (*M. canis*)
TERG_05615	-2.55	hsp70-like protein (*T. equinum*)
TERG_01562	-2.55	60S ribosomal protein L11
TERG_07786	-2.55	NAP family protein (*T. equinum*)
TERG_08353	-2.55	cytochrome P450 55A3 (*T. tonsurans*)
TERG_01609	-2.56	Na/K ATPase alpha 1 subunit (*T. equinum*)
TERG_02053	-2.56	hypothetical protein
TERG_06234	-2.56	2,5-diketo-D-gluconic acid reductase A (*T. equinum*)
TERG_04855	-2.56	hypothetical protein
TERG_05523	-2.56	hypothetical protein
TERG_11828	-2.57	hypothetical protein
TERG_06055	-2.57	NmrA-like family protein (*A. benhamiae*)
TERG_05202	-2.58	MFS drug efflux transporter (*T. equinum*)
TERG_01344	-2.58	60S ribosomal protein L22 (*T. tonsurans*)
TERG_03117	-2.58	kinesin family protein (*T. tonsurans*)
TERG_04308	-2.59	MFS sugar transporter (*T. tonsurans*)
TERG_08179	-2.59	tRNA pseudouridine synthase D (*T. equinum*)
TERG_11747	-2.59	hypothetical protein
TERG_07209	-2.60	GTPase binding protein Rid1 (*M. canis*)
TERG_11830	-2.61	hypothetical protein
TERG_03380	-2.62	FK506-binding protein 1A (*T. equinum*)
TERG_05222	-2.63	S-adenosylmethionine synthase
TERG_02166	-2.63	oxidoreductase (*T. equinum*)
TERG_02105	-2.63	hypothetical protein
TERG_06847	-2.63	Ras GTPase activating protein (*T. tonsurans*)
TERG_07639	-2.65	atypical/ABC1/ABC1-B protein kinase (*T. tonsurans*)
TERG_07099	-2.65	hypothetical protein
TERG_11550	-2.66	telomere and ribosome associated protein Stm1, putative (*T. verrucosum*)
TERG_12372	-2.66	multidrug resistance protein (*T. tonsurans*)
TERG_05849	-2.67	thioredoxin reductase (*T. equinum*)
TERG_07035	-2.67	hypothetical protein
TERG_04187	-2.68	hypothetical protein
TERG_06207	-2.68	proline oxidase PrnD (*T. verrucosum*)
TERG_01165	-2.69	40S ribosomal protein S10b (*T. equinum*)
TERG_01777	-2.69	GTP-binding protein rhoC
TERG_04531	-2.69	hypothetical protein
TERG_03707	-2.70	geranylgeranyl diphosphate synthase, putative (*A. benhamiae*)
TERG_04680	-2.72	hypothetical protein
TERG_07802	-2.73	siderophore biosynthesis acetylase AceI, putative (*T. verrucosum*)
TERG_05268	-2.74	hypothetical protein
TERG_08029	-2.74	HMG box protein, putative (*T. verrucosum*)
TERG_12357	-2.75	DNA repair protein Rad5 (*T. tonsurans*)
TERG_06137	-2.77	RNA binding protein (*A. benhamiae*)
TERG_04700	-2.77	hypothetical protein
TERG_06611	-2.77	hypothetical protein

TERG_04320	-2.78	hypothetical protein
TERG_12084	-2.79	hypothetical protein
TERG_01886	-2.80	hypothetical protein
TERG_03921	-2.83	hypothetical protein
TERG_07981	-2.83	hypothetical protein
TERG_04966	-2.85	hypothetical protein
TERG_03004	-2.86	3-methyl-2-oxobutanoate hydroxymethyltransferase
TERG_07588	-2.86	5-methyltetrahydropteroyltriglutamate-homocysteine S-methyltransferase
TERG_11710	-2.86	hypothetical protein
TERG_03148	-2.89	molybdenum cofactor sulfurase (*T. equinum*)
TERG_01139	-2.90	kinesin family protein (*T. tonsurans*)
TERG_07320	-2.91	hypothetical protein
TERG_06784	-2.91	transcriptional activator of ethanol catabolism AlcS (*A. benhamiae*)
TERG_08200	-2.94	hypothetical protein
TERG_00566	-2.94	hypothetical protein
TERG_08241	-2.96	cell division control protein 14 (*T. tonsurans*)
TERG_04738	-2.96	hypothetical protein
TERG_07045	-2.96	hypothetical protein
TERG_06403	-2.97	HASPIN protein kinase
TERG_07285	-2.97	hypothetical protein
TERG_01322	-2.98	NEK protein kinase (*T. tonsurans*)
TERG_06376	-2.99	hypothetical protein
TERG_05094	-2.99	hypothetical protein
TERG_02528	-2.99	ubiE/COQ5 methyltransferase, putative (*T. verrucosum*)
TERG_02845	-3.00	cercosporin toxin biosynthesis protein (*T. equinum*)
TERG_12159	-3.01	hypothetical protein
TERG_06049	-3.02	dimethylallyl tryptophan synthase, putative (*T. verrucosum*)
TERG_02279	-3.03	hypothetical protein
TERG_05419	-3.04	glycine dehydrogenase
TERG_08649	-3.04	cell division control protein (Cdc15), putative (*A. benhamiae*)
TERG_04666	-3.05	hypothetical protein
TERG_12566	-3.05	hypothetical protein
TERG_07469	-3.06	SUN domain-containing protein (*T. equinum*)
TERG_07905	-3.07	1-pyrroline-5-carboxylate dehydrogenase
TERG_08479	-3.09	kinesin family protein (*T. verrucosum*)
TERG_01659	-3.10	heat shock protein Hsp30-like, putative (*A. benhamiae*)
TERG_12391	-3.10	hypothetical protein
TERG_03217	-3.11	hypothetical protein
TERG_00429	-3.11	pisatin demethylase (*M. gypseum*)
TERG_12342	-3.13	membrane zinc transporter (*T. tonsurans*)
TERG_12313	-3.13	hypothetical protein
TERG_05141	-3.13	NCS1 nucleoside transporter (*T. equinum*)
TERG_04374	-3.18	STE/STE20/YSK protein kinase
TERG_03671	-3.18	hypothetical protein
TERG_03670	-3.19	hypothetical protein
TERG_03434	-3.25	GTP binding protein (*T. equinum*)
TERG_06235	-3.27	hypothetical protein
TERG_11506	-3.27	phosphoenolpyruvate carboxykinase [ATP]
TERG_03660	-3.28	hypothetical protein

TERG_08091	-3.31	conidial pigment biosynthesis oxidase Abr1/brown (*T. verrucosum*)
TERG_11621	-3.32	hypothetical protein
TERG_03060	-3.36	hypothetical protein
TERG_04234	-3.41	hydrophobin, putative (*T. verrucosum*)
TERG_06646	-3.42	arrestin (*T. equinum*)
TERG_07364	-3.42	oxidoreductase, zinc-binding dehydrogenase family superfamily (*T. verrucosum*)
TERG_04028	-3.43	cytochrome P450, putative (*T. verrucosum*)
TERG_06540	-3.44	glutathione transferase (*T. tonsurans*)
TERG_01731	-3.45	hypothetical protein
TERG_07570	-3.47	G-protein signaling regulator, putative (*T. verrucosum*)
TERG_07354	-3.48	hypothetical protein
TERG_12618	-3.50	hypothetical protein
TERG_04530	-3.54	hypothetical protein
TERG_03506	-3.54	hypothetical protein
TERG_12043	-3.55	beta-1,6 glucan synthetase (*T. tonsurans*)
TERG_08985	-3.56	hypothetical protein
TERG_04956	-3.57	hypothetical protein
TERG_03700	-3.58	hypothetical protein
TERG_00241	-3.59	hypothetical protein
TERG_08004	-3.60	hypothetical protein
TERG_01341	-3.61	protein-tyrosine phosphatase (*T. equinum*)
TERG_12017	-3.68	MFS sugar transporter, putative (*A. benhamiae*)
TERG_08648	-3.73	cell division control protein (Cdc15), putative (*A. benhamiae*)
TERG_01480	-3.74	MFS multidrug transporter, putative (*A. benhamiae*)
TERG_08054	-3.75	homoserine acetyltransferase (*T. tonsurans*)
TERG_00689	-3.75	AUR protein kinase
TERG_11505	-3.77	phosphoenolpyruvate carboxykinase [ATP]
TERG_08258	-3.79	hypothetical protein
TERG_07083	-3.80	hypothetical protein
TERG_00520	-3.82	hypothetical protein
TERG_12018	-3.85	MFS sugar transporter, putative (*A. benhamiae*)
TERG_07665	-3.86	hypothetical protein
TERG_11640	-3.89	hypothetical protein
TERG_03403	-3.92	hypothetical protein
TERG_03493	-3.94	hypothetical protein
TERG_08260	-3.96	subtilisin-like protease 2
TERG_00218	-3.97	C2H2 transcription factor (Swi5), putative (*A. benhamiae*)
TERG_04263	-4.01	hypothetical protein
TERG_05350	-4.03	hypothetical protein
TERG_00243	-4.05	pathogenesis associated protein Cap20, putative (*T. verrucosum*)
TERG_01406	-4.08	phospholipase D (*T. equinum*)
TERG_07108	-4.11	WD-repeat containing protein slp1 (*T. tonsurans*)
TERG_00499	-4.17	hypothetical protein
TERG_03936	-4.19	CAMK protein kinase
TERG_03105	-4.39	hypothetical protein
TERG_02671	-4.40	hypothetical protein
TERG_00500	-4.44	hypothetical protein
TERG_02278	-4.60	hypothetical protein
TERG_08806	-4.65	hypothetical protein

TERG_08097	-4.67	hypothetical protein
TERG_12035	-4.87	NB-ARC and TPR domain protein (*A. benhamiae*)
TERG_01599	-4.95	hypothetical protein
TERG_01148	-5.26	hypothetical protein
TERG_11771	-5.51	hypothetical protein

Gene expression values are expressed in log_2 fold change.

**ID**	**3h**	**12h**	**Gene Product Name**
TERG_06548	10.86	11.80	hypothetical protein
TERG_01782	8.34	9.02	hypothetical protein
TERG_04952	6.87	7.69	multidrug resistance protein (*T. equinum*)
TERG_08954	6.61	5.90	hypothetical protein
TERG_08751	5.82	6.33	ABC multidrug transporter, putative (*A. benhamiae*)
TERG_01543	5.42	7.48	s-adenosylmethionine (SAM)-dependent methyltransferase (*M. gypseum*)
TERG_08041	5.42	6.24	aminotransferase, putative (*A. benhamiae*)
TERG_04937	5.41	6.40	alpha/beta hydrolase (*T. equinum*)
TERG_07830	5.41	8.22	hypothetical protein
TERG_11650	4.97	5.91	hypothetical protein
TERG_03829	4.86	6.92	FAD binding domain-containing protein (*T. equinum*)
TERG_00010	4.76	8.72	amidase family protein (*T. verrucosum*)
TERG_06347	4.53	5.85	hypothetical protein
TERG_08283	4.46	2.17	acetamidase (*T. tonsurans*)
TERG_01783	4.26	4.90	hypothetical protein
TERG_04117	4.12	5.41	tannase, putative (*A. benhamiae*)
TERG_07465	3.93	3.62	serine peptidase, family S28, putative (*A. benhamiae*)
TERG_05023	3.86	2.95	calcium/proton exchanger
TERG_06779	3.85	4.61	hypothetical protein
TERG_08195	3.74	2.54	peptidase S41 family protein (*M. gypseum*)
TERG_05037	3.73	4.31	hypothetical protein
TERG_01280	3.73	4.55	IgE-binding protein (*A. benhamiae*)
TERG_02199	3.71	2.04	glutamate carboxypeptidase (*T. equinum*)
TERG_04950	3.60	4.48	cytochrome P450 monooxygenase (*T. equinum*)
TERG_05137	3.53	4.96	microsomal dipeptidase (*T. tonsurans*)
TERG_04951	3.51	3.38	U-box domain-containing protein (*T. equinum*)
TERG_01460	3.48	3.04	carboxylesterase (*T. equinum*)
TERG_02488	3.43	4.42	hypothetical protein
TERG_04960	3.36	1.75	glutathione S-transferase Ure2-like, putative (*A. benhamiae*)
TERG_07408	3.34	4.18	acetyltransferase, GNAT family (*A. benhamiae*)
TERG_02814	3.30	4.98	hypothetical protein
TERG_06106	3.27	6.45	sulfate permease 2 (*T. tonsurans*)
TERG_12228	3.26	5.00	hypothetical protein
TERG_11667	3.26	3.45	hypothetical protein
TERG_06466	3.26	1.93	hypothetical protein
TERG_07556	3.24	3.97	hypothetical protein
TERG_03970	3.23	5.40	DUF636 domain-containing protein (*T. equinum*)
TERG_01405	3.19	2.44	glutathione S-transferase (*T. equinum*)
TERG_05063	3.16	4.97	extracellular serine-rich protein (*T. verrucosum*)

TERG_04146	3.15	3.93	FAD/FMN-containing isoamyl alcohol oxidase MreA-like, putative (*A. benhamiae*)
TERG_03024	3.15	2.52	hypothetical protein
TERG_08235	3.14	3.26	long chain fatty alcohol oxidase (*T. tonsurans*)
TERG_05698	3.12	1.50	transesterase (LovD), putative (*T. verrucosum*)
TERG_05466	3.12	3.90	MFS transporter, putative (*T. verrucosum*)
TERG_12230	3.11	3.51	peptidase S41 family protein (*A. benhamiae*)
TERG_02993	3.09	4.40	allergenic cerato-platanin Asp F13 (*T. verrucosum*)
TERG_11731	3.05	4.19	zinc/cadmium resistance protein (*T. tonsurans*)
TERG_03963	3.05	3.20	mannosyl phosphorylinositol ceramide synthase SUR1 (*T. equinum*)
TERG_11633	3.03	4.50	hypothetical protein
TERG_03802	3.03	4.10	hypothetical protein
TERG_00389	3.01	3.41	hypothetical protein
TERG_02384	3.00	2.77	hypothetical protein
TERG_05639	3.00	2.73	hypothetical protein
TERG_12229	2.99	3.56	peptidase S41 family protein (*A. benhamiae*)
TERG_00060	2.93	3.54	GPI anchored cell wall protein, putative (*A. benhamiae*)
TERG_01572	2.89	4.25	carboxylesterase, putative (*T. verrucosum*)
TERG_01113	2.88	3.36	mitochondrial RNA splicing protein (*T. tonsurans*)
TERG_02589	2.84	4.78	formamidase (*T. equinum*)
TERG_08787	2.84	1.60	aminotransferase (*T. tonsurans*)
TERG_08700	2.83	1.79	DUF895 domain membrane protein (*T. equinum*)
TERG_04907	2.82	3.06	glyoxalase (*T. tonsurans*)
TERG_07094	2.81	3.55	hypothetical protein
TERG_02747	2.80	2.42	cytochrome P450 alkane hydroxylase (*A. benhamiae*)
TERG_02332	2.79	3.23	hypothetical protein
TERG_02572	2.78	1.77	hypothetical protein
TERG_12215	2.78	2.87	hypothetical protein
TERG_00254	2.75	1.74	peroxisomal dehydratase (*T. equinum*)
TERG_05442	2.74	1.81	hypothetical protein
TERG_11552	2.73	3.23	hypothetical protein
TERG_02532	2.72	4.90	C6 transcription factor, putative (*T. verrucosum*)
TERG_12216	2.71	3.06	hypothetical protein
TERG_07487	2.69	2.84	hypothetical protein
TERG_02664	2.68	2.26	hypothetical protein
TERG_00161	2.66	3.23	hypothetical protein
TERG_01943	2.66	2.71	1-aminocyclopropane-1-carboxylate deaminase
TERG_05418	2.64	2.67	hypothetical protein
TERG_04942	2.63	3.29	hypothetical protein
TERG_06208	2.63	3.47	DUF636 domain-containing protein (*M. gypseum*)
TERG_04953	2.62	3.54	cyclin, putative (*T. verrucosum*)
TERG_12217	2.62	2.86	hypothetical protein
TERG_06005	2.60	4.04	hypothetical protein
TERG_00244	2.59	2.42	stomatin family protein (*T. tonsurans*)
TERG_03353	2.58	5.16	endoglucanase (*T. equinum*)
TERG_07788	2.58	2.73	lysophospholipase (*T. tonsurans*)
TERG_06638	2.57	2.17	endochitinase (*T. equinum*)
TERG_03069	2.56	1.92	hypothetical protein
TERG_05450	2.55	1.72	acetyltransferase (*T. tonsurans*)

TERG_01876	2.53	4.07	short-chain dehydrogenase/reductase SDR (*T. tonsurans*)
TERG_07326	2.53	3.30	glutathione S-transferase PARB (*T. equinum*)
TERG_01467	2.53	4.44	cytochrome P450 monooxygenase, putative (*A. benhamiae*)
TERG_00013	2.53	2.98	ThiJ/PfpI family protein (*A. benhamiae*)
TERG_08539	2.51	4.42	Diacylglycerol kinase domain-containing protein (*T. equinum*)
TERG_03055	2.51	2.25	MFS transporter, putative (*T. verrucosum*)
TERG_07755	2.50	3.15	ergot alkaloid biosynthetic protein A (*T. verrucosum*)
TERG_00402	2.49	3.00	ABC multidrug transporter, putative (*T. verrucosum*)
TERG_07796	2.48	1.68	oxidoreductase, short-chain dehydrogenase/reductase family (*A. benhamiae*)
TERG_08077	2.47	2.90	hypothetical protein
TERG_12291	2.46	3.27	mitochondrial cytochrome b2 (*T. tonsurans*)
TERG_02989	2.45	2.35	cytochrome P450 oxidoreductase, putative (*T. verrucosum*)
TERG_03322	2.44	2.79	hypothetical protein
TERG_12290	2.44	3.22	mitochondrial cytochrome b2 (*T. tonsurans*)
TERG_02555	2.43	2.55	DUF636 domain containing protein (*T. equinum*)
TERG_12600	2.41	3.21	U3 small nucleolar ribonucleoprotein IMP4 (*T. tonsurans*)
TERG_03907	2.39	4.54	neutral amino acid permease (*T. tonsurans*)
TERG_00583	2.39	1.96	hypothetical protein
TERG_03027	2.35	2.06	hypothetical protein
TERG_12172	2.33	1.53	glycerol-3-phosphate dehydrogenase (*T. equinum*)
TERG_01934	2.33	2.69	C2H2 finger domain-containing protein (*T. equinum*)
TERG_05518	2.32	2.32	oxidoreductase, short chain dehydrogenase/reductase family (*A. benhamiae*)
TERG_00199	2.32	2.61	serine/threonine protein kinase
TERG_04224	2.32	3.96	ABC transporter
TERG_04399	2.31	1.81	MFS transporter, putative (*A. benhamiae*)
TERG_00162	2.30	2.26	MFS multidrug transporter, putative (*A. benhamiae*)
TERG_04521	2.30	3.15	HypA-like protein, putative (*A. benhamiae*)
TERG_08779	2.29	1.84	hypothetical protein
TERG_03390	2.28	2.10	glutation S-transferase (*T. equinum*)
TERG_04936	2.28	2.66	meiotic chromosome segregation protein (*T. equinum*)
TERG_03255	2.27	2.72	hypothetical protein
TERG_02838	2.24	2.22	hypothetical protein
TERG_02012	2.23	2.25	adenosine deaminase family protein (*A. benhamiae*)
TERG_02210	2.22	2.73	hypothetical protein
TERG_05669	2.22	3.29	homeobox transcription factor, putative (*T. verrucosum*)
TERG_03230	2.21	3.14	hypothetical protein
TERG_05199	2.20	2.70	MFS gliotoxin efflux transporter GliA (*T. verrucosum*)
TERG_04118	2.20	2.59	hypothetical protein
TERG_08229	2.20	2.82	hypothetical protein
TERG_04837	2.19	2.26	thioesterase (*T. equinum*)
TERG_06927	2.19	2.02	hypothetical protein
TERG_12368	2.18	3.73	hypothetical protein
TERG_11577	2.17	1.94	cysteinyl-tRNA synthetase (*M. gypseum*)
TERG_06507	2.17	2.24	oxidoreductase, 2OG-Fe(II) oxygenase family (*T. verrucosum*)
TERG_02610	2.16	2.12	WD repeat protein (*T. verrucosum*)
TERG_12065	2.16	4.89	hypothetical protein
TERG_05091	2.16	2.16	DUF636 domain-containing protein (*T. equinum*)
TERG_01042	2.14	2.91	C6 transcription factor, putative (*T. verrucosum*)
TERG_02520	2.13	2.17	DNA helicase (*T. equinum*)

TERG_11570	2.12	3.31	hypothetical protein
TERG_02212	2.12	2.79	short chain dehydrogenase/reductase family protein (*T. verrucosum*)
TERG_08360	2.12	4.15	metallo-beta-lactamase superfamily protein (*T. equinum*)
TERG_01956	2.12	3.61	C2H2 finger domain protein, putative (*A. benhamiae*)
TERG_06407	2.11	1.95	protein kinase subdomain-containing protein (*M. canis*)
TERG_02899	2.11	3.50	hypothetical protein
TERG_02566	2.10	2.93	hypothetical protein
TERG_06547	2.07	2.90	glycerophosphoryl diester phosphodiesterase (*T. tonsurans*)
TERG_00955	2.07	1.62	ABC drug exporter AtrF (*T. verrucosum*)
TERG_11801	2.06	1.70	serine/threonine protein kinase (*T. equinum*)
TERG_07134	2.05	2.67	serine/threonine protein kinase
TERG_03785	2.04	2.77	isochorismatase family hydrolase, putative (*T. verrucosum*)
TERG_06690	2.04	3.80	RTA1 domain protein, putative (*A. benhamiae*)
TERG_06990	2.04	2.81	hypothetical protein
TERG_08756	2.04	1.94	cercosporin toxin biosynthesis protein (*T. equinum*)
TERG_08300	2.03	3.95	branched-chain amino acid aminotransferase
TERG_12158	2.02	2.65	hypothetical protein
TERG_02062	2.02	1.98	hypothetical protein
TERG_05551	2.01	3.61	hypothetical protein
TERG_07428	2.00	1.73	C6 transcription factor (UaY), putative (*T. verrucosum*)
TERG_02116	2.00	2.96	PX domain-containing protein (*T. tonsurans*)
TERG_04696	2.00	1.72	MHYT domain signaling protein, putative (*T. verrucosum*)
TERG_01077	2.00	1.79	metallo-beta-lactamase domain-containing protein (*T. equinum*)
TERG_05497	1.99	2.77	C6 zinc finger domain-containing protein (*T. equinum*)
TERG_08298	1.99	3.14	RTA1 domain-containing protein (*T. equinum*)
TERG_02728	1.99	2.05	3-hydroxyisobutyrate dehydrogenase
TERG_00616	1.98	5.04	potassium/sodium efflux P-type ATPase, fungal-type
TERG_01172	1.98	2.37	hypothetical protein
TERG_04356	1.97	2.20	amino acid permease, putative (*A. benhamiae*)
TERG_11861	1.97	2.07	DNA repair helicase (rad3) (*T. tonsurans*)
TERG_02661	1.96	1.86	hypothetical protein
TERG_03253	1.96	3.57	hypothetical protein
TERG_12064	1.96	4.56	hypothetical protein
TERG_05104	1.96	1.67	MFS transporter of unknown specificity (*T. verrucosum*)
TERG_08299	1.96	3.30	multidrug resistance-associated protein 1 transporter (*M. canis*)
TERG_04509	1.94	3.55	hypothetical protein
TERG_05556	1.94	1.73	hypothetical protein
TERG_02635	1.94	3.83	hypothetical protein
TERG_12286	1.94	2.06	hypothetical protein
TERG_07787	1.94	2.28	nitrogen metabolite repression protein nmrA
TERG_04695	1.94	1.75	hypothetical protein
TERG_07198	1.92	1.92	DNA polymerase iota (*T. tonsurans*)
TERG_08050	1.92	2.16	acetamidase (*T. equinum*)
TERG_01919	1.92	2.96	serine/threonine protein kinase
TERG_02447	1.92	3.39	sulfate adenylyltransferase
TERG_04034	1.92	2.86	hypothetical protein
TERG_00914	1.92	2.71	hypothetical protein
TERG_11804	1.92	2.05	hypothetical protein
TERG_03229	1.92	1.65	hexokinase (*T. tonsurans*)

TERG_02186	1.91	3.47	ABC multidrug transporter, putative (*A. benhamiae*)
TERG_02704	1.91	2.68	short-chain dehydrogenase/reductase, putative (*A. benhamiae*)
TERG_06369	1.90	2.40	PQ loop repeat protein (*A. benhamiae*)
TERG_02112	1.90	2.69	hypothetical protein
TERG_01594	1.88	2.35	hypothetical protein
TERG_02449	1.88	3.75	phosphoadenosine phosphosulfate reductase
TERG_05492	1.87	2.39	hypothetical protein
TERG_05134	1.87	2.35	O-methyltransferase GliM (*A. benhamiae*)
TERG_11782	1.87	3.36	hypothetical protein
TERG_00574	1.86	3.07	MFS multidrug transporter, putative (*T. verrucosum*)
TERG_00127	1.86	2.81	secretory phospholipase A2 (*T. tonsurans*)
TERG_04763	1.85	2.01	fructosyl amine:oxygen oxidoreductase (*T. tonsurans*)
TERG_07847	1.85	3.04	hypothetical protein
TERG_12083	1.85	2.21	hypothetical protein
TERG_01964	1.85	1.60	cystathionine gamma-lyase (*T. equinum*)
TERG_11832	1.84	4.18	cytosolic Fe-S cluster assembling factor NBP35 (*T. tonsurans*)
TERG_07647	1.83	1.55	hypothetical protein
TERG_12287	1.83	2.27	hypothetical protein
TERG_05456	1.83	2.28	hypothetical protein
TERG_02311	1.83	2.92	hypothetical protein
TERG_04174	1.83	1.81	citrinin biosynthesis oxydoreductase CtnB (*T. equinum*)
TERG_12066	1.82	2.01	AMP-binding enzyme, putative (*T. verrucosum*)
TERG_06004	1.82	2.61	hypothetical protein
TERG_01986	1.81	3.40	Fe-S protein assembly co-chaperone HscB
TERG_03771	1.80	2.47	ankyrin repeat protein (*T. verrucosum*)
TERG_12654	1.79	2.16	hypothetical protein
TERG_08503	1.79	2.51	secalin (*M. canis*)
TERG_00523	1.78	3.29	hypothetical protein
TERG_03892	1.78	1.62	CAMK/CAMKL protein kinase (*T. tonsurans*)
TERG_03815	1.78	1.75	subtilisin-like protease 3
TERG_01925	1.77	3.53	hypothetical protein
TERG_02565	1.77	1.82	sulfate transporter (*T. equinum*)
TERG_12583	1.76	2.34	hypothetical protein
TERG_12044	1.75	2.90	hypothetical protein
TERG_08568	1.75	2.47	CMGC/SRPK protein kinase
TERG_11792	1.75	1.72	hypothetical protein
TERG_06377	1.75	1.63	metalloproteinase, putative (*A. benhamiae*)
TERG_02920	1.75	2.98	RTA1 domain-containing protein (*T. equinum*)
TERG_05459	1.74	2.89	hypothetical protein
TERG_05824	1.74	2.46	hypothetical protein
TERG_03331	1.72	1.75	F-box protein (*T. equinum*)
TERG_03333	1.72	2.26	transcription factor RfeD, putative (*A. benhamiae*)
TERG_08181	1.72	2.14	hypothetical protein
TERG_07859	1.72	3.02	ubiquitin C-terminal hydrolase L3 (*T. tonsurans*)
TERG_01319	1.71	2.68	hypothetical protein
TERG_12213	1.71	1.59	hypothetical protein
TERG_01468	1.71	1.60	hypothetical protein
TERG_12385	1.71	3.03	hypothetical protein
TERG_03312	1.70	3.03	RTA1 domain protein, putative (*A. benhamiae*)

TERG_08286	1.70	1.53	GPI anchored CFEM domain protein (*T. verrucosum*)
TERG_02593	1.69	2.21	C2H2 finger domain protein, putative (*T. verrucosum*)
TERG_00528	1.69	1.75	hypothetical protein
TERG_04769	1.69	1.85	extracellular serine carboxypeptidase, putative (*T. verrucosum*)
TERG_08654	1.69	1.84	hypothetical protein
TERG_04766	1.67	2.07	calcium/proton exchanger
TERG_06792	1.67	1.88	hypothetical protein
TERG_12099	1.66	2.38	hypothetical protein
TERG_08451	1.65	1.51	hypothetical protein
TERG_01459	1.64	2.60	conserved leucine-rich repeat protein (*T. verrucosum*)
TERG_05198	1.64	1.67	hypothetical protein
TERG_03240	1.64	1.70	transmembrane efflux protein (*T. tonsurans*)
TERG_12067	1.63	1.97	AMP-binding enzyme, putative (*T. verrucosum*)
TERG_12327	1.63	3.18	alkaline serine protease (PR1)/allergen F18, putative (*T. verrucosum*)
TERG_03291	1.63	4.41	SNF2 family helicase, putative (*T. verrucosum*)
TERG_11949	1.63	2.65	hypothetical protein
TERG_06105	1.63	2.66	hypothetical protein
TERG_12320	1.62	1.83	hypothetical protein
TERG_01636	1.62	2.54	ADP-ribosylglycohydrolase (*M. gypseum*)
TERG_08178	1.62	2.14	endoglucanase (*T. equinum*)
TERG_03078	1.61	1.77	cytochrome P450 oxidoreductase OrdA-like, putative (*T. verrucosum*)
TERG_06740	1.61	2.48	hypothetical protein
TERG_04504	1.61	3.97	extracellular protein (*T. equinum*)
TERG_05421	1.60	2.11	alpha-galactosidase A (*M. canis*)
TERG_08351	1.60	2.50	hypothetical protein
TERG_12591	1.59	2.88	peptidase S8 family protein (*T. equinum*)
TERG_01637	1.59	3.74	hypothetical protein
TERG_07964	1.59	2.20	vacuolar endopolyphosphatase, putative (*T. verrucosum*)
TERG_03330	1.59	1.84	hypothetical protein
TERG_08781	1.59	1.97	hypothetical protein
TERG_02847	1.59	1.95	Fasciclin domain family protein (*T. verrucosum*)
TERG_02995	1.58	2.27	hypothetical protein
TERG_08825	1.58	1.93	bZIP transcription factor JlbA/IDI-4 (*T. verrucosum*)
TERG_01082	1.57	1.66	hypothetical protein
TERG_04194	1.57	2.99	acyltransferase (*T. tonsurans*)
TERG_01266	1.57	2.26	amidophosphoribosyltransferase
TERG_02794	1.57	2.46	hypothetical protein
TERG_08324	1.56	1.88	hypothetical protein
TERG_02147	1.56	2.36	3-demethylubiquinone-9 3-methyltransferase, putative (*T. verrucosum*)
TERG_07443	1.56	2.17	hypothetical protein
TERG_02059	1.55	1.74	AbpA protein (*T. equinum*)
TERG_12032	1.55	2.36	hypothetical protein
TERG_02848	1.55	1.54	short chain dehydrogenase/reductase family protein (*A. benhamiae*)
TERG_03884	1.55	-2.04	hypothetical protein
TERG_06065	1.55	4.07	conserved glycine-rich protein (*T. verrucosum*)
TERG_07095	1.55	1.90	hypothetical protein
TERG_04954	1.55	4.09	hypothetical protein
TERG_00575	1.54	1.61	F-box domain protein (*T. verrucosum*)
TERG_01623	1.54	2.98	MFS transporter (*T. equinum*)

TERG_02900	1.53	3.12	hypothetical protein
TERG_06650	1.53	1.63	MFS monocarboxylate transporter, putative (*A. benhamiae*)
TERG_06147	1.53	1.50	pyridine nucleotide-disulfide oxidoreductase, putative (*A. benhamiae*)
TERG_07352	1.53	4.12	aldehyde reductase (AKR1), putative (*T. verrucosum*)
TERG_00384	1.52	1.58	Ser/Thr protein phosphatase family protein (*A. benhamiae*)
TERG_12640	1.52	2.29	choriogenin Hminor (*M. canis*)
TERG_02185	1.52	1.99	hypothetical protein
TERG_04245	1.52	1.73	hypothetical protein
TERG_12002	1.51	1.61	hypothetical protein
TERG_01895	1.51	2.33	pheromone processing carboxypeptidase (Sxa2), putative (*T. verrucosum*)
TERG_07197	1.50	3.02	disintegrin and metalloproteinase domain-containing protein B
TERG_05303	1.50	1.88	phospholipase PldA, putative (*T. verrucosum*)
TERG_05374	1.50	2.35	hypothetical protein
TERG_02489	1.50	2.02	sister chromatid cohesion acetyltransferase Eco1, putative (*A. benhamiae*)
TERG_11724	-1.51	-3.66	polyketide synthase, putative (*A. benhamiae*)
TERG_04742	-1.52	-5.29	hypothetical protein
TERG_06187	-1.52	-1.84	nucleolar ATPase Kre33 (*T. tonsurans*)
TERG_00507	-1.53	-3.57	hypothetical protein
TERG_12319	-1.53	-2.81	chitin synthase 2
TERG_02327	-1.53	-2.63	rRNA biogenesis protein RRP5 (*T. tonsurans*)
TERG_00569	-1.55	-3.66	C2H2 finger domain protein, putative (*A. benhamiae*)
TERG_03278	-1.55	-2.57	hypothetical protein
TERG_02425	-1.55	-2.44	RNA binding protein (*A. benhamiae*)
TERG_01012	-1.56	-2.03	hypothetical protein
TERG_00342	-1.56	-2.02	phosphoglucomutase
TERG_04791	-1.56	-1.58	hypothetical protein
TERG_04558	-1.57	-2.48	nucleoside diphosphate kinase
TERG_05819	-1.57	-1.99	isocitrate dehydrogenase (*T. tonsurans*)
TERG_11986	-1.58	-3.34	GABA permease, putative (*A. benhamiae*)
TERG_02623	-1.61	-3.91	hypothetical protein
TERG_11987	-1.61	-3.90	GABA permease, putative (*A. benhamiae*)
TERG_01883	-1.61	-3.23	hsp75-like protein
TERG_05717	-1.61	-3.10	squalene epoxidase (*T. equinum*)
TERG_04857	-1.61	-2.85	ribose 5-phosphate isomerase
TERG_11689	-1.63	-1.97	U3 small nucleolar RNA-associated protein 10 (*T. equinum*)
TERG_01911	-1.64	-3.59	NmrA family protein (*T. equinum*)
TERG_07048	-1.65	-2.42	dsDNA-dependent ATPase (*T. equinum*)
TERG_00189	-1.65	-2.87	40S ribosomal protein S0
TERG_01202	-1.68	-2.11	histone H3-like centromeric protein cnp1
TERG_04041	-1.69	-2.21	Sad1/UNC domain protein (*T. verrucosum*)
TERG_05435	-1.70	-1.62	hypothetical protein
TERG_07351	-1.71	-2.81	hypothetical protein
TERG_00760	-1.71	-3.40	protein arginine methyltransferase (*T. tonsurans*)
TERG_08139	-1.71	-2.67	NAD dependent epimerase/dehydratase (*M. gypseum*)
TERG_02353	-1.73	-3.00	UbiD family decarboxylase, putative (*T. verrucosum*)
TERG_01002	-1.74	-1.89	chaperone dnaK (*T. tonsurans*)
TERG_00253	-1.74	-2.77	HNRNP arginine N-methyltransferase (*T. tonsurans*)
TERG_12232	-1.75	-2.69	DUF221 domain-containing protein (*T. equinum*)
TERG_03175	-1.75	-3.97	DUF833 domain protein (*A. benhamiae*)

TERG_00657	-1.77	-2.38	hypothetical protein
TERG_08680	-1.77	-2.05	amino acid permease (*T. tonsurans*)
TERG_00694	-1.77	-3.65	glutamate 5-kinase
TERG_05780	-1.78	-2.25	hypothetical protein
TERG_07200	-1.78	-2.48	C2 domain-containing protein (*T. tonsurans*)
TERG_11785	-1.78	-1.83	hypothetical protein
TERG_01252	-1.78	-3.32	catalase A
TERG_01062	-1.78	-3.08	hypothetical protein
TERG_11852	-1.80	-2.22	glyoxalase family protein (*T. verrucosum*)
TERG_02114	-1.83	-4.27	glycerol dehydrogenase (*T. tonsurans*)
TERG_12193	-1.84	-2.38	MFS sugar transporter (*T. equinum*)
TERG_12156	-1.87	-3.31	hypothetical protein
TERG_05585	-1.94	-2.99	short-chain dehydrogenase/reductase SDR (*T. equinum*)
TERG_01273	-1.97	-3.29	alpha-1,2-mannosidase, putative subfamily (*T. verrucosum*)
TERG_00567	-1.98	-2.76	hypothetical protein
TERG_12194	-2.01	-2.53	MFS monosaccharide transporter, putative (*T. verrucosum*)
TERG_05808	-2.02	-3.25	hypothetical protein
TERG_02081	-2.03	-3.19	RNA binding protein
TERG_11963	-2.06	-4.79	hypothetical protein
TERG_00855	-2.08	-3.48	aromatic amino acid aminotransferase, putative (*A. benhamiae*)
TERG_00852	-2.08	-3.99	Phytanoyl-CoA dioxygenase PhyH (*T. equinum*)
TERG_11536	-2.09	-5.69	hypothetical protein
TERG_12038	-2.21	-2.85	protein phosphatase regulatory subunit Gac1, putative (*T. verrucosum*)
TERG_07652	-2.22	-2.16	Delta(12) fatty acid desaturase (*T. equinum*)
TERG_05139	-2.24	-2.13	non ribosomal peptide synthase (*T. equinum*)
TERG_02357	-2.24	-2.32	amino acid permease (*T. equinum*)
TERG_06715	-2.27	-2.79	LEA domain protein (*A. benhamiae*)
TERG_04282	-2.35	-4.76	GPI ethanolamine phosphate transferase (*T. tonsurans*)
TERG_03919	-2.51	-5.20	phytoene dehydrogenase (*T. equinum*)
TERG_00856	-2.54	-2.86	phytanoyl-CoA dioxygenase (*T. equinum*)
TERG_03677	-2.57	-3.11	protein phosphatase regulatory subunit Gac1, putative (*T. verrucosum*)
TERG_01917	-2.60	-2.32	O-methyltransferase, putative (*A. benhamiae*)
TERG_04580	-2.64	-3.58	NADP-specific glutamate dehydrogenase
TERG_03826	-2.77	-3.57	hypothetical protein
TERG_00490	-3.13	-5.24	erythromycin esterase (*T. tonsurans*)
TERG_01619	-3.20	-2.35	toxin biosynthesis protein (Tri7), putative (*T. verrucosum*)
TERG_12339	-3.31	-2.95	hypothetical protein
TERG_05816	-3.74	-1.95	hypothetical protein
TERG_02959	-4.16	-5.90	hypothetical protein
TERG_02653	-6.21	-2.23	hypothetical protein

Gene expression values are expressed in $\log_2$ fold change.
